# RepeatFS: a file system providing reproducibility through provenance and automation

**DOI:** 10.1093/bioinformatics/btaa950

**Published:** 2020-11-24

**Authors:** Anthony Westbrook, Elizabeth Varki, W Kelley Thomas

**Affiliations:** Department of Computer Science; Hubbard Center for Genome Studies; Department of Computer Science; Hubbard Center for Genome Studies; Department of Molecular Cellular and Biomedical Sciences, University of New Hampshire, Durham, NH 03824, USA

## Abstract

**Motivation:**

Reproducibility is of central importance to the scientific process. The difficulty of consistently replicating and verifying experimental results is magnified in the era of big data, in which bioinformatics analysis often involves complex multi-application pipelines operating on terabytes of data. These processes result in thousands of possible permutations of data preparation steps, software versions and command-line arguments. Existing reproducibility frameworks are cumbersome and involve redesigning computational methods. To address these issues, we developed RepeatFS, a file system that records, replicates and verifies informatics workflows with no alteration to the original methods. RepeatFS also provides several other features to help promote analytical transparency and reproducibility, including provenance visualization and task automation.

**Results:**

We used RepeatFS to successfully visualize and replicate a variety of bioinformatics tasks consisting of over a million operations with no alteration to the original methods. RepeatFS correctly identified all software inconsistencies that resulted in replication differences.

**Availabilityand implementation:**

RepeatFS is implemented in Python 3. Its source code and documentation are available at https://github.com/ToniWestbrook/repeatfs.

**Supplementary information:**

Supplementary data are available at *Bioinformatics* online.

## 1 Introduction

The foundation of science is built upon the acquisition and analysis of empirical evidence. The formulation and testing of hypotheses are rooted in these observations. This methodology provides the capability not only to understand the world around us but also to make effective predictions given specific conditions. As such, accurate forecasts require a reproducible experimental design; repetition of a deterministic process with differing results indicates a lack of rigor and eliminates the confidence in the prediction. Given the importance of repeatability, the enterprise of science currently faces a significant struggle. A 2016 *Nature* survey completed by over 1500 researchers found that more than 70% have attempted to reproduce another scientist’s findings without success (Baker, 2016, 500), and more than half were unable to replicate their own results. Subsequently, numerous supporting studies have been performed across a range of biological disciplines, including genomics (Kanwal *et al.*, 2017), biomedical sciences (Coiera *et al.*, 2018) and computational biology (Garijo *et al.*, 2013), each noting challenges involved in ensuring reproducibility.

While many factors in the research process can result in a deviation from the original experimental methods, each study notes informatics as especially problematic, often responsible for introducing unintended variation into replication studies. Respondents of the *Nature* survey corroborate this concern, with over 82% noting that ‘insufficient computer code or protocol information’ is at least sometimes involved, if not very often or always. Reasons for this stem from the vast number of available software applications and reference databases (Davis-Turak *et al.*, 2017); differences in versions, parameters and configuration files (Kim et al., 2018); and a wide variety of data formats and conversion techniques (Lewis et al., 2016). As a typical informatics workflow has the potential for thousands of combinations of these attributes, it quickly becomes apparent that provenance for recording the exact environment and steps performed by a researcher is critical in replicating results.

Informatics software has attempted to address this reproducibility issue with limited success. Virtual environments, such as Docker (Docker, 2020) and Anaconda (Anaconda Software Distribution, 2020), ensure the versions of software match between original and repeated analyses, but cannot verify these programs are executed using the same parameters, reference databases or other runtime options. Analysis platforms such as Galaxy (Afgan *et al.*, 2018) and QIIME 2 (Bolyen *et al.*, 2019) require the researcher to operate within the provided set of tools, forcing methods to be redesigned and precluding the ability to use many external packages. Generic pipeline frameworks such as Ruffus (Goodstadt, 2010), Bpipe (Sadedin et al., 2012), Snakemake (Köster et al., 2012) and SoS (Wang et al., 2019), while designed to work with any program, require the researcher to write scripts to migrate their workflow into the framework. This not only requires learning an additional language and rewriting each step of the workflow but potentially introduces new mistakes. Thus, current solutions have limitations that prevent their widespread adoption.

To provide robust provenance capabilities without requiring the user to learn new platforms or languages, we developed RepeatFS, a file system that transparently records process, file and read/write activity for every application. Informatics methods may be used without alteration, and the complete provenance history of any file may be exported, visualized and replicated. Replication supports process verification, ensuring the same parameters, software versions and resulting files are produced for each step in the provenance record, including provenance histories with multiple versions of the same application. RepeatFS also has the ability to reconstruct script files, should these be missing or unavailable during the replication process.

In addition to these provenance services, RepeatFS provides ‘virtual dynamic files’ (VDFs) for commonly performed informatics tasks, such as converting between file formats or filtering delimited tables. VDFs are displayed in a directory listing as normal files, though they do not reside on disk. For each file matching a file type designated by the user, a corresponding VDF will be shown in the directory listing. This VDF will be named the same as the source file but will end in a new extension that indicates the type of data the VDF contains. When the VDF is accessed, RepeatFS automatically runs one or more pre-configured programs, such as a file conversion utility, using the source file as input. The output of this program is streamed into the VDF. An example configuration would show a FASTA VDF for each FASTQ file present on disk. If opened, the FASTA file would contain the sequences from the FASTQ file with the quality scores removed; RepeatFS automatically runs the conversion utility when the FASTA file is accessed. This process ensures these tasks are always performed in an identical manner and reduces the risk of error. Though VDFs are created in this unique way, the system treats them as normal files, and they may be viewed, copied and used as input for other applications.

By offering both provenance tracking and VDFs, RepeatFS reduces the risk of user error and promotes reproducibility. RepeatFS is written in Python 3, supports all major underlying single and multiuser file systems (ext4, Lustre, BeeGFS) and is user installable on Linux (soon available for MacOS). RepeatFS does not currently support recording provenance across different file systems.

## 2 Materials and methods

To demonstrate the effectiveness of RepeatFS, we established two primary goals. The first was to run two common bioinformatics pipelines on a source computer system, then visualize, replicate and verify the results on a mix of destination server environments with differing levels of similarity to the source. The second goal was to demonstrate a variety of common tasks using VDFs. Widely used open-source software was utilized for all tests, and no application employed their own provenance tracking capabilities.

### 2.1 Evaluating provenance tracking

The reproduction of a target file requires the exact replicated execution of every program that created or modified that file. All files previously read by those programs must also be reproduced, and this process must be recursively repeated until the entire program tree has been re-created. For this reason, the foundation of the RepeatFS replication system relies upon a complete and detailed historical provenance record of every file operation performed on all monitored files. In order to effectively test and demonstrate this functionality, two bioinformatics pipelines were constructed and used. These pipelines were limited in scope to aid verification and visualization but contained a large volume of different file operations to create a complex provenance history. No such restrictions are required during normal use of RepeatFS.

The first pipeline performed genome annotation using the *SRA toolkit*(Leinonen et al., 2011) for obtaining reads, *Trimmomatic* (Bolger *et al.*, 2014) for adapter and quality trimming, *SPAdes* (Bankevich *et al.*, 2012) for genome assembly and *Prokka* (Seemann, 2014) for genome annotation. Nearly one million, paired-end reads of *E.coli* were used as input. The resulting GFF file was used as the target file for replication. The second pipeline performed phylogenetic inference of a 16S gene tree using *wget* to obtain the SILVA (Quast et al., 2012) reference database, *bioawk* (Li, 2011) to filter the reference for entries belonging to a particular taxonomic rank, *MAFFT* (Katoh, 2002) for multi-sequence alignment and *RAxML* (Stamatakis, 2014) for maximum-likelihood based phylogenetic inference. The SILVA reference was filtered for the 718 present members of the Yersinia genus. The resulting Newick file was used as the target file for replication.

After executing each pipeline on the source server environment *EnvSrc*, provenance was exported for each target file. Each export file was copied to two destination server environments, *EnvDst1* and *EnvDst2*. Neither pipeline shell script was copied from *EnvSrc*. *EnvDst1* contained identical versions of all software as *EnvSrc*, while *EnvDst2* was modified to contain different versions of software used by each pipeline: *BLAST* (Altschul *et al.*, 1990), a well-known application used by *Prokka*, was upgraded to a later version, and RAxML was recompiled with different default options. The exported provenance was then imported into RepeatFS to perform pipeline script reconstruction, replication and result verification. As a secondary confirmation, the resulting target file was compared using *diff* to the original to note any inconsistencies.

### 2.2 Evaluating virtual dynamic files

As the purpose of VDFs is to automate commonly executed commands, two classes of tests were designed to demonstrate the effectiveness of VDFs across a variety of bioinformatics tasks. The first class defined VDFs for converting genomics file formats and instructed RepeatFS to provide a corresponding converted file for each original file in a directory. This configuration included VDF FASTA files for FASTQ files via FASTX-Toolkit (Gordon, 2014), VDF sorted BAM files for SAM files via Samtools (Li *et al.*, 2009) and VDF Phylip (Felsenstein) files for Clustal (Thompson et al., 1994) files via BioPython (Cock *et al.*, 2009). The second class defined VDFs for analysis tasks and included FASTA and SAM file parsing routines. These VDFs provided FASTA headers via grep, alignment statistics via Samtools, and taxon abundance via cut, sort and uniq. Though by no means an exhaustive list of possible functions VDFs can provide, these examples illustrate a representative sample of typical bioinformatics tasks.

## 3 Results

Following the execution of both pipelines within EnvSrc, we calculated and noted metrics relevant to the size and complexity of the provenance as recorded by RepeatFS (Table 1). Though only comprised of fewer than 10 applications, together both pipelines yielded nearly 1.5 million IO operations. The number of total documented command-line parameters available across all applications was nearly 400. Lastly, the number of different versions and releases that have been publicly available during the lifetime of each application was estimated at over 400.

**Table 1.** Provenance complexity

Executable	IO Ops	Parameters	Releases
Fastq-dump	268 552	43	48
Trimmomatic	261 775	17	39
SPAdes	609 644	53	32
Prokka	39194	39	25
***Pipeline 1 total***	**1 179 165**	**152**	**144**
Wget	96 449	148	35
GZip	202 196	18	12
Bioawk	6547	Many*	1
MAFFT	1496	8	177
RAxML	2356	69	68
***Pipeline 2 total***	**309 044**	**243**	**293**
***Combined total***	**1 488 209**	**395**	**437**

IO operations were recorded as any direct request by an application against the RepeatFS file system (e.g. open, read, write), as well as operations performed indirectly by the operating system as a result of these requests. Application parameter counts only include those explicitly documented by the usage output. As Bioawk extends Awk, a domain-specific language with all commands available as parameters, this number was not included in calculations. Releases were estimated using changelogs, version control repository releases (Github) and numbering systems. Releases were confined to the application only and did not include linked libraries. Each pipeline was executed under amd64 Debian and CentOS Linux.

**Fig. 1.**
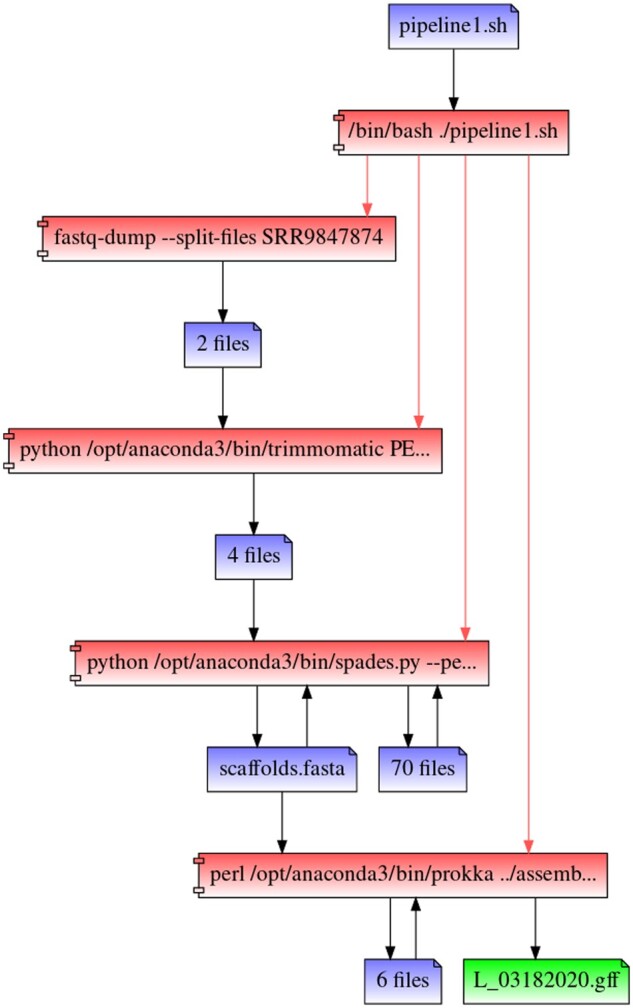
A provenance graph was generated by RepeatFS for the target annotation file (green) for pipeline 1. Relationships between processes (red) and files (blue) are shown for every causal read or write operation (black arrows) that affected the creation or modification of the target file. Each pipeline shell script was expanded to display spawned child processes (red arrows). Files with identical read and write processes are automatically grouped and counted, greatly reducing the visual complexity of the graph. Though top-level graphs are shown here, we were also able to further expand and verify sub-process activity under parent programs, such as SPAdes and Prokka

**Fig. 2.**
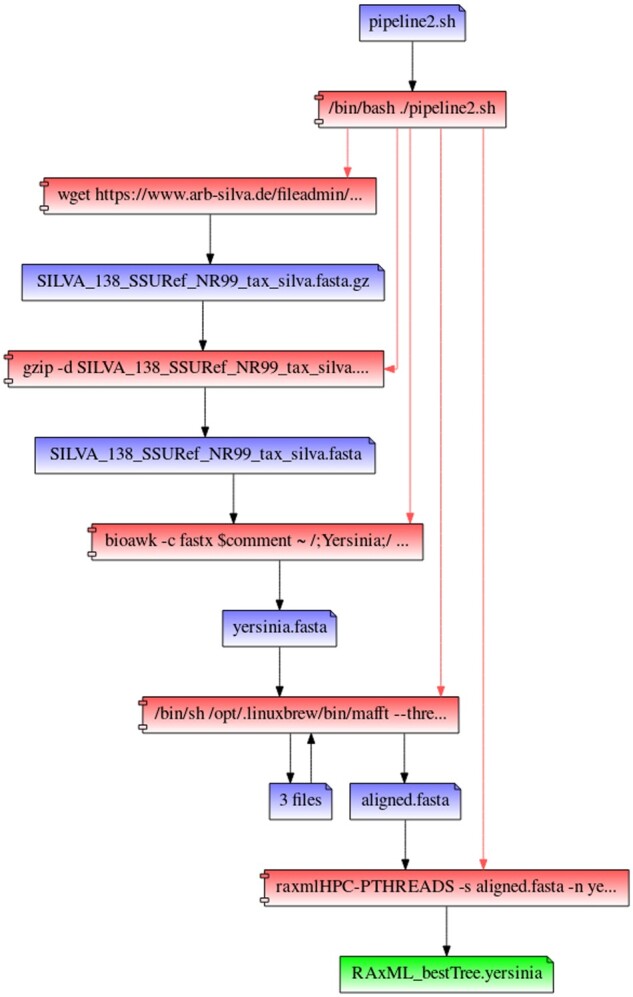
A provenance graph was generated by RepeatFS for the target tree file (green) for pipeline 2. Relationships between processes (red) and files (blue) are shown for every causal read or write operation (black arrows) that affected the creation or modification of the target file. Each pipeline shell script was expanded to display spawned child processes (red arrows). Files with identical read and write processes are automatically grouped and counted, greatly reducing the visual complexity of the graph.

Utilizing RepeatFS provenance visualization, we manually confirmed the accuracy of the process execution and file access records (Figs 1 and 2). Each shell script process was expanded to examine the activity of all child processes and ensure each application executed as expected. RepeatFS successfully recorded all operations and correctly grouped files with identical read/write activity.

Following visualization, we used the exported provenance from *EnvSrc* for each target file as the basis of replication in *EnvDst1* and *EnvDst2*. RepeatFS was able to successfully reconstruct and execute the original pipeline scripts in both environments, and noted software version differences in *BLAST* and *RAxML* in *EnvDst2*. Comparing the resulting target files for each pipeline revealed that *EnvSrc* and *EnvDst1* yielded identical results, while the output from *EnvDst2* contained differences for both pipelines caused by the difference in software versions.

Finally, the contents of the six VDFs were compared with the results created by manually running the commands associated with each VDF definition; in all cases, the two files were identical. In addition, each VDF was copied into a separate directory outside of RepeatFS, confirming the ability to export the results of these files to an outside environment.

## 4 Implementation

### 4.1 Functional overview

**Fig. 3.**
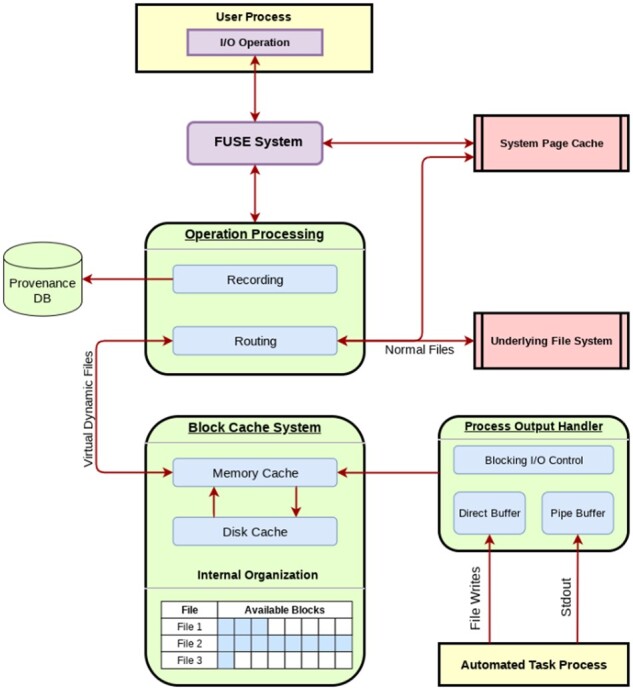
RepeatFS structure, outlining the flow of data originating from a system call issued by a process. The system call is first directed into RepeatFS by FUSE. Once the operation is received, information necessary to later reconstruct provenance is stored within a database and then sent for routing. Operations performed on real files are relayed to the underlying file system, and those performed on VDFs are handled by the block cache system. Since RepeatFS is a multithreaded file system, multiple system calls and VDF task processes may be serviced concurrently

To provide a universal and reliable method of recording provenance, we elected to implement RepeatFS as a file system, providing the ability to monitor all I/O operations. This avoids reconstructing provenance through incomplete and ambiguous information derived from command history logs, process lists or environment variables. RepeatFS utilizes the popular FUSE interface, allowing the user to run one or more instances without requiring system administrator privileges. When accessing disk files, RepeatFS acts as an interface, receiving applicable system calls, relaying them to the underlying file system and returning the results (see Operation Processing in Fig. 3).

### 4.2 VDF processing

Many bioinformatics applications are designed to write output to a stdout stream so the user may utilize a pipe and relay the results to a receiving program. This allows the receiving program to immediately begin processing output before the sending program has finished execution. Using these standard memory-based POSIX pipes is limited however, as the data are lost once the receiving program reads it. Multiple programs may not simultaneously receive the same data from a pipe, and the sending program must be run again with a new pipe for each repeated execution.

For programs that write directly to disk files instead of stdout streams, these issues are solved as multiple applications may read the output file concurrently. However, the application reading the data is normally unaware of the total amount to read. While pipes indicate if all data have been sent, disk files cannot provide this indication and all data must be written to a file before other programs may safely access it. The performance improvement of a receiving program concurrently processing the data are therefore lost. In addition, temporary disk files must be used, decreasing performance and increasing unnecessary file clutter.

RepeatFS solves the limitations of both pipes and disk files by providing VDFs. System calls made to VDFs are not relayed to the underlying file system and are instead routed through RepeatFS’s custom block cache system, reducing the number of disk accesses (see *Block Cache System* in Fig. 3). When a process issues a read request, if the requested block is present in the memory cache, the data are immediately retrieved. If the block is present in the disk cache, it is transferred to the memory cache and then returned. In the event of the first cache miss, RepeatFS executes the shell command to populate the VDF as defined in the configuration file. These commands may provide output via a stdout stream or writes to a file, and the user may configure which destination to receive. RepeatFS will route this output into a buffer and transfer the requested data to the appropriate location in the block memory cache, thereby populating the missing block (see *Process Output Handler* in Fig. 3). Each subsequent cache miss for the file will cause RepeatFS to transfer additional output to the next location within the block memory cache until the application ends and no further output remains. Should the memory cache size ever exceed the configured maximum, RepeatFS will flush the oldest, unwritten blocks out to the disk cache.

Since VDFs are presented as disk files and not pipes, multiple receiving programs may access them concurrently. Unlike normal disk files however, RepeatFS will pause any reads made to blocks not yet written by the sending program; once the block is written, the read will resume, allowing receiving programs to begin reading data before the sending program has finished. This hybrid approach offers the benefits of both pipe and disk-based file access.

### 4.3 Storing and retrieving provenance

After receiving a system call for either disk files or VDFs, RepeatFS records the operation within a SQL database, noting the time it occurred, the file targeted by the operation and details about the process issuing the call. This information includes attributes necessary to subsequently reconstruct provenance, including command-line parameters, current working directory and the executable’s checksum. Operation records associated with high throughput operations such as reads and writes are temporarily cached in memory and later flushed to the database to improve performance.

When visualizing or replicating the provenance of a file of interest, RepeatFS iteratively traces through I/O activity within the database by repeatedly executing two queries. The first query retrieves all the past write operations made to the file, noting the execution details of each process that performed a write. For each of these processes, the second query retrieves any read operations made to other files by the process prior to the time the process wrote to the file of interest, since it is likely the data obtained from these reads were used as input for the internal routines that wrote to the file of interest. A simple example of this would be a sorting program which reads words from an input file and then writes a sorted list of these words to an output file; the words written are dependent on the words read. In lieu of a computationally expensive analysis of machine code to determine dependence, RepeatFS instead assumes data written by a process is dependent on all data read earlier by the process.

Finally, each file targeted by these reads then becomes the start of a new iteration, and the entire process is continually repeated. An additional constraint ensures that only files written before the second query’s read time are included in the next iteration. This recursively reconstructs the provenance tree while accounting for temporal correctness; only data read by a process prior to the time the process produced output could have affected this output. Recursion will end once no further processes are associated with any provenance branch. The earliest file in a provenance branch was created by a process that used input data from outside of RepeatFS, such as from another file system, a network location or the keyboard.

## 5 Implementation

As shown with the example pipelines, the number of parameters and versions of the component applications create thousands of potential runtime combinations, each of which may lead to a different resulting output. Though the pipelines consisted of only nine total applications, the combined number of file system operations was over one million, illustrating that the number of applications utilized in a pipeline is a poor predictor of provenance size or complexity. RepeatFS does not limit the numbers of applications or file operations used in a pipeline.

Though some attributes associated with execution variability, such as parameters and configuration files, may be faithfully reproduced using careful notetaking, others are potentially unknown to the user. Applications may reference internal values or proprietary data files, each of which may vary between versions. Table 1 illustrates the magnitude of software versions and file access counts for a small selection of bioinformatics applications, but this issue is inherent within all software. This problem clearly demonstrates the need for a record of provenance at the file system level, as well as an automated way of replicating and verifying the large number of potential execution variations.

By using the provenance graphs produced by RepeatFS, we were able to easily ascertain many details about the inner components of each pipeline that could potentially cause differing outputs in future executions. We were also able to automatically replicate the results of both complex pipelines with complete fidelity when run using an environment identical to the original, even without access to the original pipeline shell scripts. When run in a modified environment, RepeatFS was able to correctly warn of the potential for different results due to the mismatch in software versions. Performing the same level of verification manually would not only require arduous amounts of work but also require detailed technical knowledge of each component in the pipeline, creating the potential for human error. In addition, each of the six tasks we performed using VDFs resulted in identical results, showing this feature reduces workload for repeatedly run tasks and strengthens reproducibility.

Most importantly, we were able to accomplish these replication and verification tasks easily without the need to write scripts or modify our bioinformatics methods to fit within a custom framework. Thus, RepeatFS avoids introducing variability and errors into a pipeline caused by misconfiguring the workflow management system. As the correct replication of results is still dependent on matching software versions, we recommend utilizing RepeatFS with software managed by a virtual or container environment, such as Anaconda or Docker. When used in this manner, RepeatFS is an easy-to-use tool for ensuring reproducibility for virtually any type of informatics analysis.

## Funding

This work was supported by New Hampshire-INBRE through an Institutional Development Award (IDeA), P20GM103506, from the National Institute of General Medical Sciences of the NIH.

*Conflict of Interest*: none declared.

## Supplementary Material

btaa950_Supplementary_Data
