# Supplementary material for: RepeatFS: a file system providing reproducibility through provenance and automation
Source: Bioinformatics. 2020 Nov 24;37(9):1292–6. doi: 10.1093/bioinformatics/btaa950 (PMC8189677; doi:10.1093/bioinformatics/btaa950)
Supplement: btaa950_Supplementary_Data [file btaa950_supplementary_data.docx]

RepeatFS Supplementary

# Pipeline script: Genome annotation

#! /bin/bash

touch pipeline1-1.txt

mkdir reads

cd reads

fastq-dump --split-files SRR9847874

touch ../pipeline1-2.txt

trimmomatic PE SRR9847874_1.fastq SRR9847874_2.fastq out_fwd_paired.fq out_fwd_unpaired.fq out_rev_paired.fq out_rev_unpaired.fq ILLUMINACLIP:TruSeq3-PE.fa:2:30:10 LEADING:3 TRAILING:3 SLIDINGWINDOW:4:15 MINLEN:36 -threads 6

touch ../pipeline1-3.txt

cd ..

mkdir assembly

cd assembly

spades.py --pe1-1 ../reads/out_fwd_paired.fq --pe1-2 ../reads/out_rev_paired.fq --pe1-s ../reads/out_fwd_unpaired.fq --pe1-s ../reads/out_rev_unpaired.fq -k 77 --tmp-dir /tmp/spades -o output

touch ../pipeline1-4.txt

cd ..

mkdir annotation

cd annotation

prokka ../assembly/output/scaffolds.fasta --centre C --locustag L --force

# Pipeline script: Phylogenetic inference

#! /bin/bash

touch pipeline2-1.txt

mkdir reference

cd reference

wget <https://www.arb->silva.de/fileadmin/silva_databases/current/Exports/SILVA_138_SSURef_NR99_tax_silva.fasta.gz

touch pipeline2-2.txt

gzip -d SILVA_138_SSURef_NR99_tax_silva.fasta.gz

touch pipeline2-3.txt

bioawk -c fastx '$comment ~ /;Yersinia;/ { print ">" $name " " $comment "\n" $seq }' SILVA_138_SSURef_NR99_tax_silva.fasta > yersinia.fasta

touch pipeline2-4.txt

cd ..

mkdir tree

cd tree

mafft --thread 8 ../reference/yersinia.fasta > aligned.fasta

touch pipeline2-5.txt

raxmlHPC-PTHREADS -s aligned.fasta -n yersinia -m GTRCAT -T 8 -p 1

# Genome annotation verification – EnvDst1 (width truncated)

[info] Starting replication

[info] Replication complete

[info] Starting verification

[ok] Process 28486 (mkdir reads) executed

[ok] Process 28490 (fastq-dump --split-files SRR9847874) executed

[ok] Process 28528 (python /home/anthonyw/anaconda3/bin/trimmomatic PE SRR9847874_1.fastq SRR9847874_2.fastq out_fwd_pai

[ok] Process 28601 (mkdir assembly) executed

[ok] Process 28605 (python /home/anthonyw/anaconda3/bin/spades.py --pe1-1 ../reads/out_fwd_paired.fq --pe1-2 ../reads/ou

[ok] Process 30826 (mkdir annotation) executed

[ok] Process 30830 (perl /home/anthonyw/anaconda3/bin/prokka ../assembly/output/scaffolds.fasta --centre C --locustag L

[ok] Process 28532 (java -Xms512m -Xmx1g -jar /home/anthonyw/anaconda3/share/trimmomatic-0.39-1/trimmomatic.jar PE SRR98

[ok] Process 28533 (java -Xms512m -Xmx1g -jar /home/anthonyw/anaconda3/share/trimmomatic-0.39-1/trimmomatic.jar PE SRR98

[ok] Process 28555 (java -Xms512m -Xmx1g -jar /home/anthonyw/anaconda3/share/trimmomatic-0.39-1/trimmomatic.jar PE SRR98

[ok] Process 28556 (java -Xms512m -Xmx1g -jar /home/anthonyw/anaconda3/share/trimmomatic-0.39-1/trimmomatic.jar PE SRR98

[ok] Process 28557 (java -Xms512m -Xmx1g -jar /home/anthonyw/anaconda3/share/trimmomatic-0.39-1/trimmomatic.jar PE SRR98

[ok] Process 28558 (java -Xms512m -Xmx1g -jar /home/anthonyw/anaconda3/share/trimmomatic-0.39-1/trimmomatic.jar PE SRR98

[ok] Process 28611 (/home/anthonyw/anaconda3/share/spades-3.13.1-0/bin/spades-hammer $$$/assembly/output/corrected/confi

[ok] Process 29254 (python /home/anthonyw/anaconda3/bin/spades.py --pe1-1 ../reads/out_fwd_paired.fq --pe1-2 ../reads/ou

[ok] Process 29255 (python /home/anthonyw/anaconda3/bin/spades.py --pe1-1 ../reads/out_fwd_paired.fq --pe1-2 ../reads/ou

[ok] Process 29256 (python /home/anthonyw/anaconda3/bin/spades.py --pe1-1 ../reads/out_fwd_paired.fq --pe1-2 ../reads/ou

[ok] Process 29257 (python /home/anthonyw/anaconda3/bin/spades.py --pe1-1 ../reads/out_fwd_paired.fq --pe1-2 ../reads/ou

[ok] Process 29258 (python /home/anthonyw/anaconda3/bin/spades.py --pe1-1 ../reads/out_fwd_paired.fq --pe1-2 ../reads/ou

[ok] Process 29318 (/home/anthonyw/anaconda3/share/spades-3.13.1-0/bin/spades-core $$$/assembly/output/K77/configs/confi

[ok] Process 30835 (mkdir -p PROKKA_03292020) executed

[ok] Process 30969 (sh -c cat PROKKA_03292020\/PROKKA_03292020\.IS\.tmp\.30830\.faa | parallel --gnu --tmpdir $$$/tmp --

[ok] Process 31083 (sh -c cat PROKKA_03292020\/PROKKA_03292020\.AMR\.tmp\.30830\.faa | parallel --gnu --tmpdir $$$/tmp -

[ok] Process 31197 (sh -c cat PROKKA_03292020\/PROKKA_03292020\.sprot\.tmp\.30830\.faa | parallel --gnu --tmpdir $$$/tmp

[ok] Process 31340 (sh -c cat PROKKA_03292020\/PROKKA_03292020\.HAMAP\.hmm\.tmp\.30830\.faa | parallel --gnu --tmpdir $$

[ok] Process 32632 (sh -c tbl2asn -V b -a r10k -l paired-ends -M n -N 1 -y 'Annotated using prokka 1.14.6 from https://g

[ok] Process 29263 (gzip -f -7 $$$/assembly/output/corrected/out_fwd_paired.00.0_0.cor.fastq) executed

[ok] Process 29262 (gzip -f -7 $$$/assembly/output/corrected/out_rev_paired.00.0_0.cor.fastq) executed

[ok] Process 29264 (gzip -f -7 $$$/assembly/output/corrected/out__unpaired.00.0_0.cor.fastq) executed

[ok] Process 29266 (gzip -f -7 $$$/assembly/output/corrected/out_fwd_unpaired.00.0_1.cor.fastq) executed

[ok] Process 29265 (gzip -f -7 $$$/assembly/output/corrected/out_rev_unpaired.00.0_2.cor.fastq) executed

[ok] Process 30970 (cat PROKKA_03292020/PROKKA_03292020.IS.tmp.30830.faa) executed

[ok] Process 30971 (perl /home/anthonyw/anaconda3/bin/parallel --gnu --tmpdir $$$/tmp --plain -j 8 --block 94265 --recst

[ok] Process 31084 (cat PROKKA_03292020/PROKKA_03292020.AMR.tmp.30830.faa) executed

[ok] Process 31085 (perl /home/anthonyw/anaconda3/bin/parallel --gnu --tmpdir $$$/tmp --plain -j 8 --block 93546 --recst

[ok] Process 31198 (cat PROKKA_03292020/PROKKA_03292020.sprot.tmp.30830.faa) executed

[ok] Process 31199 (perl /home/anthonyw/anaconda3/bin/parallel --gnu --tmpdir $$$/tmp --plain -j 8 --block 93546 --recst

[ok] Process 31341 (cat PROKKA_03292020/PROKKA_03292020.HAMAP.hmm.tmp.30830.faa) executed

[ok] Process 31342 (perl /home/anthonyw/anaconda3/bin/parallel --gnu --tmpdir $$$/tmp --plain -j 8 --block 19274 --recst

[ok] Process 32633 (tbl2asn -V b -a r10k -l paired-ends -M n -N 1 -y Annotated using prokka 1.14.6 from https://github.c

[ok] Process 30983 (/bin/sh -c perl -e 'if(sysread(STDIN,$buf,1)){open($fh,"|-",@ARGV)||die;syswrite($fh,$buf);if($read=

[ok] Process 30985 (/bin/sh -c perl -e 'if(sysread(STDIN,$buf,1)){open($fh,"|-",@ARGV)||die;syswrite($fh,$buf);if($read=

[ok] Process 30987 (/bin/sh -c perl -e 'if(sysread(STDIN,$buf,1)){open($fh,"|-",@ARGV)||die;syswrite($fh,$buf);if($read=

[ok] Process 30989 (/bin/sh -c perl -e 'if(sysread(STDIN,$buf,1)){open($fh,"|-",@ARGV)||die;syswrite($fh,$buf);if($read=

[ok] Process 30991 (/bin/sh -c perl -e 'if(sysread(STDIN,$buf,1)){open($fh,"|-",@ARGV)||die;syswrite($fh,$buf);if($read=

[ok] Process 30993 (/bin/sh -c perl -e 'if(sysread(STDIN,$buf,1)){open($fh,"|-",@ARGV)||die;syswrite($fh,$buf);if($read=

[ok] Process 30995 (/bin/sh -c perl -e 'if(sysread(STDIN,$buf,1)){open($fh,"|-",@ARGV)||die;syswrite($fh,$buf);if($read=

[ok] Process 30997 (/bin/sh -c perl -e 'if(sysread(STDIN,$buf,1)){open($fh,"|-",@ARGV)||die;syswrite($fh,$buf);if($read=

[ok] Process 31031 (/bin/sh -c perl -e 'if(sysread(STDIN,$buf,1)){open($fh,"|-",@ARGV)||die;syswrite($fh,$buf);if($read=

[ok] Process 31036 (/bin/sh -c perl -e 'if(sysread(STDIN,$buf,1)){open($fh,"|-",@ARGV)||die;syswrite($fh,$buf);if($read=

[ok] Process 31038 (/bin/sh -c perl -e 'if(sysread(STDIN,$buf,1)){open($fh,"|-",@ARGV)||die;syswrite($fh,$buf);if($read=

[ok] Process 31043 (/bin/sh -c perl -e 'if(sysread(STDIN,$buf,1)){open($fh,"|-",@ARGV)||die;syswrite($fh,$buf);if($read=

[ok] Process 31048 (/bin/sh -c perl -e 'if(sysread(STDIN,$buf,1)){open($fh,"|-",@ARGV)||die;syswrite($fh,$buf);if($read=

[ok] Process 31050 (/bin/sh -c perl -e 'if(sysread(STDIN,$buf,1)){open($fh,"|-",@ARGV)||die;syswrite($fh,$buf);if($read=

[ok] Process 31055 (/bin/sh -c perl -e 'if(sysread(STDIN,$buf,1)){open($fh,"|-",@ARGV)||die;syswrite($fh,$buf);if($read=

[ok] Process 31066 (/bin/sh -c perl -e 'if(sysread(STDIN,$buf,1)){open($fh,"|-",@ARGV)||die;syswrite($fh,$buf);if($read=

[ok] Process 31075 (/bin/sh -c perl -e 'if(sysread(STDIN,$buf,1)){open($fh,"|-",@ARGV)||die;syswrite($fh,$buf);if($read=

[ok] Process 31097 (/bin/sh -c perl -e 'if(sysread(STDIN,$buf,1)){open($fh,"|-",@ARGV)||die;syswrite($fh,$buf);if($read=

[ok] Process 31099 (/bin/sh -c perl -e 'if(sysread(STDIN,$buf,1)){open($fh,"|-",@ARGV)||die;syswrite($fh,$buf);if($read=

[ok] Process 31101 (/bin/sh -c perl -e 'if(sysread(STDIN,$buf,1)){open($fh,"|-",@ARGV)||die;syswrite($fh,$buf);if($read=

[ok] Process 31103 (/bin/sh -c perl -e 'if(sysread(STDIN,$buf,1)){open($fh,"|-",@ARGV)||die;syswrite($fh,$buf);if($read=

[ok] Process 31105 (/bin/sh -c perl -e 'if(sysread(STDIN,$buf,1)){open($fh,"|-",@ARGV)||die;syswrite($fh,$buf);if($read=

[ok] Process 31107 (/bin/sh -c perl -e 'if(sysread(STDIN,$buf,1)){open($fh,"|-",@ARGV)||die;syswrite($fh,$buf);if($read=

[ok] Process 31109 (/bin/sh -c perl -e 'if(sysread(STDIN,$buf,1)){open($fh,"|-",@ARGV)||die;syswrite($fh,$buf);if($read=

[ok] Process 31111 (/bin/sh -c perl -e 'if(sysread(STDIN,$buf,1)){open($fh,"|-",@ARGV)||die;syswrite($fh,$buf);if($read=

[ok] Process 31145 (/bin/sh -c perl -e 'if(sysread(STDIN,$buf,1)){open($fh,"|-",@ARGV)||die;syswrite($fh,$buf);if($read=

[ok] Process 31150 (/bin/sh -c perl -e 'if(sysread(STDIN,$buf,1)){open($fh,"|-",@ARGV)||die;syswrite($fh,$buf);if($read=

[ok] Process 31153 (/bin/sh -c perl -e 'if(sysread(STDIN,$buf,1)){open($fh,"|-",@ARGV)||die;syswrite($fh,$buf);if($read=

[ok] Process 31161 (/bin/sh -c perl -e 'if(sysread(STDIN,$buf,1)){open($fh,"|-",@ARGV)||die;syswrite($fh,$buf);if($read=

[ok] Process 31163 (/bin/sh -c perl -e 'if(sysread(STDIN,$buf,1)){open($fh,"|-",@ARGV)||die;syswrite($fh,$buf);if($read=

[ok] Process 31165 (/bin/sh -c perl -e 'if(sysread(STDIN,$buf,1)){open($fh,"|-",@ARGV)||die;syswrite($fh,$buf);if($read=

[ok] Process 31170 (/bin/sh -c perl -e 'if(sysread(STDIN,$buf,1)){open($fh,"|-",@ARGV)||die;syswrite($fh,$buf);if($read=

[ok] Process 31178 (/bin/sh -c perl -e 'if(sysread(STDIN,$buf,1)){open($fh,"|-",@ARGV)||die;syswrite($fh,$buf);if($read=

[ok] Process 31189 (/bin/sh -c perl -e 'if(sysread(STDIN,$buf,1)){open($fh,"|-",@ARGV)||die;syswrite($fh,$buf);if($read=

[ok] Process 31211 (/bin/sh -c perl -e 'if(sysread(STDIN,$buf,1)){open($fh,"|-",@ARGV)||die;syswrite($fh,$buf);if($read=

[ok] Process 31213 (/bin/sh -c perl -e 'if(sysread(STDIN,$buf,1)){open($fh,"|-",@ARGV)||die;syswrite($fh,$buf);if($read=

[ok] Process 31215 (/bin/sh -c perl -e 'if(sysread(STDIN,$buf,1)){open($fh,"|-",@ARGV)||die;syswrite($fh,$buf);if($read=

[ok] Process 31217 (/bin/sh -c perl -e 'if(sysread(STDIN,$buf,1)){open($fh,"|-",@ARGV)||die;syswrite($fh,$buf);if($read=

[ok] Process 31219 (/bin/sh -c perl -e 'if(sysread(STDIN,$buf,1)){open($fh,"|-",@ARGV)||die;syswrite($fh,$buf);if($read=

[ok] Process 31221 (/bin/sh -c perl -e 'if(sysread(STDIN,$buf,1)){open($fh,"|-",@ARGV)||die;syswrite($fh,$buf);if($read=

[ok] Process 31223 (/bin/sh -c perl -e 'if(sysread(STDIN,$buf,1)){open($fh,"|-",@ARGV)||die;syswrite($fh,$buf);if($read=

[ok] Process 31225 (/bin/sh -c perl -e 'if(sysread(STDIN,$buf,1)){open($fh,"|-",@ARGV)||die;syswrite($fh,$buf);if($read=

[ok] Process 31270 (/bin/sh -c perl -e 'if(sysread(STDIN,$buf,1)){open($fh,"|-",@ARGV)||die;syswrite($fh,$buf);if($read=

[ok] Process 31275 (/bin/sh -c perl -e 'if(sysread(STDIN,$buf,1)){open($fh,"|-",@ARGV)||die;syswrite($fh,$buf);if($read=

[ok] Process 31280 (/bin/sh -c perl -e 'if(sysread(STDIN,$buf,1)){open($fh,"|-",@ARGV)||die;syswrite($fh,$buf);if($read=

[ok] Process 31288 (/bin/sh -c perl -e 'if(sysread(STDIN,$buf,1)){open($fh,"|-",@ARGV)||die;syswrite($fh,$buf);if($read=

[ok] Process 31290 (/bin/sh -c perl -e 'if(sysread(STDIN,$buf,1)){open($fh,"|-",@ARGV)||die;syswrite($fh,$buf);if($read=

[ok] Process 31295 (/bin/sh -c perl -e 'if(sysread(STDIN,$buf,1)){open($fh,"|-",@ARGV)||die;syswrite($fh,$buf);if($read=

[ok] Process 31300 (/bin/sh -c perl -e 'if(sysread(STDIN,$buf,1)){open($fh,"|-",@ARGV)||die;syswrite($fh,$buf);if($read=

[ok] Process 31302 (/bin/sh -c perl -e 'if(sysread(STDIN,$buf,1)){open($fh,"|-",@ARGV)||die;syswrite($fh,$buf);if($read=

[ok] Process 31329 (/bin/sh -c perl -e 'if(sysread(STDIN,$buf,1)){open($fh,"|-",@ARGV)||die;syswrite($fh,$buf);if($read=

[ok] Process 31354 (/bin/sh -c perl -e 'if(sysread(STDIN,$buf,1)){open($fh,"|-",@ARGV)||die;syswrite($fh,$buf);if($read=

[ok] Process 31356 (/bin/sh -c perl -e 'if(sysread(STDIN,$buf,1)){open($fh,"|-",@ARGV)||die;syswrite($fh,$buf);if($read=

[ok] Process 31358 (/bin/sh -c perl -e 'if(sysread(STDIN,$buf,1)){open($fh,"|-",@ARGV)||die;syswrite($fh,$buf);if($read=

[ok] Process 31360 (/bin/sh -c perl -e 'if(sysread(STDIN,$buf,1)){open($fh,"|-",@ARGV)||die;syswrite($fh,$buf);if($read=

[ok] Process 31362 (/bin/sh -c perl -e 'if(sysread(STDIN,$buf,1)){open($fh,"|-",@ARGV)||die;syswrite($fh,$buf);if($read=

[ok] Process 31364 (/bin/sh -c perl -e 'if(sysread(STDIN,$buf,1)){open($fh,"|-",@ARGV)||die;syswrite($fh,$buf);if($read=

[ok] Process 31366 (/bin/sh -c perl -e 'if(sysread(STDIN,$buf,1)){open($fh,"|-",@ARGV)||die;syswrite($fh,$buf);if($read=

[ok] Process 31368 (/bin/sh -c perl -e 'if(sysread(STDIN,$buf,1)){open($fh,"|-",@ARGV)||die;syswrite($fh,$buf);if($read=

[ok] Process 31879 (/bin/sh -c perl -e 'if(sysread(STDIN,$buf,1)){open($fh,"|-",@ARGV)||die;syswrite($fh,$buf);if($read=

[ok] Process 31910 (/bin/sh -c perl -e 'if(sysread(STDIN,$buf,1)){open($fh,"|-",@ARGV)||die;syswrite($fh,$buf);if($read=

[ok] Process 31945 (/bin/sh -c perl -e 'if(sysread(STDIN,$buf,1)){open($fh,"|-",@ARGV)||die;syswrite($fh,$buf);if($read=

[ok] Process 31978 (/bin/sh -c perl -e 'if(sysread(STDIN,$buf,1)){open($fh,"|-",@ARGV)||die;syswrite($fh,$buf);if($read=

[ok] Process 31989 (/bin/sh -c perl -e 'if(sysread(STDIN,$buf,1)){open($fh,"|-",@ARGV)||die;syswrite($fh,$buf);if($read=

[ok] Process 32007 (/bin/sh -c perl -e 'if(sysread(STDIN,$buf,1)){open($fh,"|-",@ARGV)||die;syswrite($fh,$buf);if($read=

[ok] Process 32027 (/bin/sh -c perl -e 'if(sysread(STDIN,$buf,1)){open($fh,"|-",@ARGV)||die;syswrite($fh,$buf);if($read=

[ok] Process 32047 (/bin/sh -c perl -e 'if(sysread(STDIN,$buf,1)){open($fh,"|-",@ARGV)||die;syswrite($fh,$buf);if($read=

[ok] Process 32265 (/bin/sh -c perl -e 'if(sysread(STDIN,$buf,1)){open($fh,"|-",@ARGV)||die;syswrite($fh,$buf);if($read=

[ok] Process 30984 (perl -e if(sysread(STDIN,$buf,1)){open($fh,"|-",@ARGV)||die;syswrite($fh,$buf);if($read=sysread(STDI

[ok] Process 30986 (perl -e if(sysread(STDIN,$buf,1)){open($fh,"|-",@ARGV)||die;syswrite($fh,$buf);if($read=sysread(STDI

[ok] Process 30988 (perl -e if(sysread(STDIN,$buf,1)){open($fh,"|-",@ARGV)||die;syswrite($fh,$buf);if($read=sysread(STDI

[ok] Process 30990 (perl -e if(sysread(STDIN,$buf,1)){open($fh,"|-",@ARGV)||die;syswrite($fh,$buf);if($read=sysread(STDI

[ok] Process 30992 (perl -e if(sysread(STDIN,$buf,1)){open($fh,"|-",@ARGV)||die;syswrite($fh,$buf);if($read=sysread(STDI

[ok] Process 30994 (perl -e if(sysread(STDIN,$buf,1)){open($fh,"|-",@ARGV)||die;syswrite($fh,$buf);if($read=sysread(STDI

[ok] Process 30996 (perl -e if(sysread(STDIN,$buf,1)){open($fh,"|-",@ARGV)||die;syswrite($fh,$buf);if($read=sysread(STDI

[ok] Process 31019 (perl -e if(sysread(STDIN,$buf,1)){open($fh,"|-",@ARGV)||die;syswrite($fh,$buf);if($read=sysread(STDI

[ok] Process 31033 (perl -e if(sysread(STDIN,$buf,1)){open($fh,"|-",@ARGV)||die;syswrite($fh,$buf);if($read=sysread(STDI

[ok] Process 31040 (perl -e if(sysread(STDIN,$buf,1)){open($fh,"|-",@ARGV)||die;syswrite($fh,$buf);if($read=sysread(STDI

[ok] Process 31044 (perl -e if(sysread(STDIN,$buf,1)){open($fh,"|-",@ARGV)||die;syswrite($fh,$buf);if($read=sysread(STDI

[ok] Process 31051 (perl -e if(sysread(STDIN,$buf,1)){open($fh,"|-",@ARGV)||die;syswrite($fh,$buf);if($read=sysread(STDI

[ok] Process 31057 (perl -e if(sysread(STDIN,$buf,1)){open($fh,"|-",@ARGV)||die;syswrite($fh,$buf);if($read=sysread(STDI

[ok] Process 31060 (perl -e if(sysread(STDIN,$buf,1)){open($fh,"|-",@ARGV)||die;syswrite($fh,$buf);if($read=sysread(STDI

[ok] Process 31063 (perl -e if(sysread(STDIN,$buf,1)){open($fh,"|-",@ARGV)||die;syswrite($fh,$buf);if($read=sysread(STDI

[ok] Process 31068 (perl -e if(sysread(STDIN,$buf,1)){open($fh,"|-",@ARGV)||die;syswrite($fh,$buf);if($read=sysread(STDI

[ok] Process 31077 (perl -e if(sysread(STDIN,$buf,1)){open($fh,"|-",@ARGV)||die;syswrite($fh,$buf);if($read=sysread(STDI

[ok] Process 31098 (perl -e if(sysread(STDIN,$buf,1)){open($fh,"|-",@ARGV)||die;syswrite($fh,$buf);if($read=sysread(STDI

[ok] Process 31100 (perl -e if(sysread(STDIN,$buf,1)){open($fh,"|-",@ARGV)||die;syswrite($fh,$buf);if($read=sysread(STDI

[ok] Process 31102 (perl -e if(sysread(STDIN,$buf,1)){open($fh,"|-",@ARGV)||die;syswrite($fh,$buf);if($read=sysread(STDI

[ok] Process 31104 (perl -e if(sysread(STDIN,$buf,1)){open($fh,"|-",@ARGV)||die;syswrite($fh,$buf);if($read=sysread(STDI

[ok] Process 31106 (perl -e if(sysread(STDIN,$buf,1)){open($fh,"|-",@ARGV)||die;syswrite($fh,$buf);if($read=sysread(STDI

[ok] Process 31108 (perl -e if(sysread(STDIN,$buf,1)){open($fh,"|-",@ARGV)||die;syswrite($fh,$buf);if($read=sysread(STDI

[ok] Process 31110 (perl -e if(sysread(STDIN,$buf,1)){open($fh,"|-",@ARGV)||die;syswrite($fh,$buf);if($read=sysread(STDI

[ok] Process 31135 (perl -e if(sysread(STDIN,$buf,1)){open($fh,"|-",@ARGV)||die;syswrite($fh,$buf);if($read=sysread(STDI

[ok] Process 31147 (perl -e if(sysread(STDIN,$buf,1)){open($fh,"|-",@ARGV)||die;syswrite($fh,$buf);if($read=sysread(STDI

[ok] Process 31155 (perl -e if(sysread(STDIN,$buf,1)){open($fh,"|-",@ARGV)||die;syswrite($fh,$buf);if($read=sysread(STDI

[ok] Process 31158 (perl -e if(sysread(STDIN,$buf,1)){open($fh,"|-",@ARGV)||die;syswrite($fh,$buf);if($read=sysread(STDI

[ok] Process 31167 (perl -e if(sysread(STDIN,$buf,1)){open($fh,"|-",@ARGV)||die;syswrite($fh,$buf);if($read=sysread(STDI

[ok] Process 31171 (perl -e if(sysread(STDIN,$buf,1)){open($fh,"|-",@ARGV)||die;syswrite($fh,$buf);if($read=sysread(STDI

[ok] Process 31175 (perl -e if(sysread(STDIN,$buf,1)){open($fh,"|-",@ARGV)||die;syswrite($fh,$buf);if($read=sysread(STDI

[ok] Process 31180 (perl -e if(sysread(STDIN,$buf,1)){open($fh,"|-",@ARGV)||die;syswrite($fh,$buf);if($read=sysread(STDI

[ok] Process 31183 (perl -e if(sysread(STDIN,$buf,1)){open($fh,"|-",@ARGV)||die;syswrite($fh,$buf);if($read=sysread(STDI

[ok] Process 31191 (perl -e if(sysread(STDIN,$buf,1)){open($fh,"|-",@ARGV)||die;syswrite($fh,$buf);if($read=sysread(STDI

[ok] Process 31212 (perl -e if(sysread(STDIN,$buf,1)){open($fh,"|-",@ARGV)||die;syswrite($fh,$buf);if($read=sysread(STDI

[ok] Process 31214 (perl -e if(sysread(STDIN,$buf,1)){open($fh,"|-",@ARGV)||die;syswrite($fh,$buf);if($read=sysread(STDI

[ok] Process 31216 (perl -e if(sysread(STDIN,$buf,1)){open($fh,"|-",@ARGV)||die;syswrite($fh,$buf);if($read=sysread(STDI

[ok] Process 31218 (perl -e if(sysread(STDIN,$buf,1)){open($fh,"|-",@ARGV)||die;syswrite($fh,$buf);if($read=sysread(STDI

[ok] Process 31220 (perl -e if(sysread(STDIN,$buf,1)){open($fh,"|-",@ARGV)||die;syswrite($fh,$buf);if($read=sysread(STDI

[ok] Process 31222 (perl -e if(sysread(STDIN,$buf,1)){open($fh,"|-",@ARGV)||die;syswrite($fh,$buf);if($read=sysread(STDI

[ok] Process 31224 (perl -e if(sysread(STDIN,$buf,1)){open($fh,"|-",@ARGV)||die;syswrite($fh,$buf);if($read=sysread(STDI

[ok] Process 31249 (perl -e if(sysread(STDIN,$buf,1)){open($fh,"|-",@ARGV)||die;syswrite($fh,$buf);if($read=sysread(STDI

[ok] Process 31272 (perl -e if(sysread(STDIN,$buf,1)){open($fh,"|-",@ARGV)||die;syswrite($fh,$buf);if($read=sysread(STDI

[ok] Process 31277 (perl -e if(sysread(STDIN,$buf,1)){open($fh,"|-",@ARGV)||die;syswrite($fh,$buf);if($read=sysread(STDI

[ok] Process 31285 (perl -e if(sysread(STDIN,$buf,1)){open($fh,"|-",@ARGV)||die;syswrite($fh,$buf);if($read=sysread(STDI

[ok] Process 31292 (perl -e if(sysread(STDIN,$buf,1)){open($fh,"|-",@ARGV)||die;syswrite($fh,$buf);if($read=sysread(STDI

[ok] Process 31297 (perl -e if(sysread(STDIN,$buf,1)){open($fh,"|-",@ARGV)||die;syswrite($fh,$buf);if($read=sysread(STDI

[ok] Process 31303 (perl -e if(sysread(STDIN,$buf,1)){open($fh,"|-",@ARGV)||die;syswrite($fh,$buf);if($read=sysread(STDI

[ok] Process 31307 (perl -e if(sysread(STDIN,$buf,1)){open($fh,"|-",@ARGV)||die;syswrite($fh,$buf);if($read=sysread(STDI

[ok] Process 31309 (perl -e if(sysread(STDIN,$buf,1)){open($fh,"|-",@ARGV)||die;syswrite($fh,$buf);if($read=sysread(STDI

[ok] Process 31334 (perl -e if(sysread(STDIN,$buf,1)){open($fh,"|-",@ARGV)||die;syswrite($fh,$buf);if($read=sysread(STDI

[ok] Process 31355 (perl -e if(sysread(STDIN,$buf,1)){open($fh,"|-",@ARGV)||die;syswrite($fh,$buf);if($read=sysread(STDI

[ok] Process 31357 (perl -e if(sysread(STDIN,$buf,1)){open($fh,"|-",@ARGV)||die;syswrite($fh,$buf);if($read=sysread(STDI

[ok] Process 31359 (perl -e if(sysread(STDIN,$buf,1)){open($fh,"|-",@ARGV)||die;syswrite($fh,$buf);if($read=sysread(STDI

[ok] Process 31361 (perl -e if(sysread(STDIN,$buf,1)){open($fh,"|-",@ARGV)||die;syswrite($fh,$buf);if($read=sysread(STDI

[ok] Process 31363 (perl -e if(sysread(STDIN,$buf,1)){open($fh,"|-",@ARGV)||die;syswrite($fh,$buf);if($read=sysread(STDI

[ok] Process 31365 (perl -e if(sysread(STDIN,$buf,1)){open($fh,"|-",@ARGV)||die;syswrite($fh,$buf);if($read=sysread(STDI

[ok] Process 31367 (perl -e if(sysread(STDIN,$buf,1)){open($fh,"|-",@ARGV)||die;syswrite($fh,$buf);if($read=sysread(STDI

[ok] Process 31401 (perl -e if(sysread(STDIN,$buf,1)){open($fh,"|-",@ARGV)||die;syswrite($fh,$buf);if($read=sysread(STDI

[ok] Process 31948 (perl -e if(sysread(STDIN,$buf,1)){open($fh,"|-",@ARGV)||die;syswrite($fh,$buf);if($read=sysread(STDI

[ok] Process 31952 (perl -e if(sysread(STDIN,$buf,1)){open($fh,"|-",@ARGV)||die;syswrite($fh,$buf);if($read=sysread(STDI

[ok] Process 31968 (perl -e if(sysread(STDIN,$buf,1)){open($fh,"|-",@ARGV)||die;syswrite($fh,$buf);if($read=sysread(STDI

[ok] Process 32056 (perl -e if(sysread(STDIN,$buf,1)){open($fh,"|-",@ARGV)||die;syswrite($fh,$buf);if($read=sysread(STDI

[ok] Process 32098 (perl -e if(sysread(STDIN,$buf,1)){open($fh,"|-",@ARGV)||die;syswrite($fh,$buf);if($read=sysread(STDI

[ok] Process 32128 (perl -e if(sysread(STDIN,$buf,1)){open($fh,"|-",@ARGV)||die;syswrite($fh,$buf);if($read=sysread(STDI

[ok] Process 32168 (perl -e if(sysread(STDIN,$buf,1)){open($fh,"|-",@ARGV)||die;syswrite($fh,$buf);if($read=sysread(STDI

[ok] Process 32185 (perl -e if(sysread(STDIN,$buf,1)){open($fh,"|-",@ARGV)||die;syswrite($fh,$buf);if($read=sysread(STDI

[ok] Process 32368 (perl -e if(sysread(STDIN,$buf,1)){open($fh,"|-",@ARGV)||die;syswrite($fh,$buf);if($read=sysread(STDI

[ok] Process 30999 (/bin/sh -c blastp -query - -db /home/anthonyw/anaconda3/db/kingdom/Bacteria/IS -evalue 1e-30 -qcov_h

[ok] Process 31001 (/bin/sh -c blastp -query - -db /home/anthonyw/anaconda3/db/kingdom/Bacteria/IS -evalue 1e-30 -qcov_h

[ok] Process 31004 (/bin/sh -c blastp -query - -db /home/anthonyw/anaconda3/db/kingdom/Bacteria/IS -evalue 1e-30 -qcov_h

[ok] Process 31009 (/bin/sh -c blastp -query - -db /home/anthonyw/anaconda3/db/kingdom/Bacteria/IS -evalue 1e-30 -qcov_h

[ok] Process 31018 (/bin/sh -c blastp -query - -db /home/anthonyw/anaconda3/db/kingdom/Bacteria/IS -evalue 1e-30 -qcov_h

[ok] Process 31011 (/bin/sh -c blastp -query - -db /home/anthonyw/anaconda3/db/kingdom/Bacteria/IS -evalue 1e-30 -qcov_h

[ok] Process 31014 (/bin/sh -c blastp -query - -db /home/anthonyw/anaconda3/db/kingdom/Bacteria/IS -evalue 1e-30 -qcov_h

[ok] Process 31021 (/bin/sh -c blastp -query - -db /home/anthonyw/anaconda3/db/kingdom/Bacteria/IS -evalue 1e-30 -qcov_h

[ok] Process 31034 (/bin/sh -c blastp -query - -db /home/anthonyw/anaconda3/db/kingdom/Bacteria/IS -evalue 1e-30 -qcov_h

[ok] Process 31041 (/bin/sh -c blastp -query - -db /home/anthonyw/anaconda3/db/kingdom/Bacteria/IS -evalue 1e-30 -qcov_h

[ok] Process 31046 (/bin/sh -c blastp -query - -db /home/anthonyw/anaconda3/db/kingdom/Bacteria/IS -evalue 1e-30 -qcov_h

[ok] Process 31053 (/bin/sh -c blastp -query - -db /home/anthonyw/anaconda3/db/kingdom/Bacteria/IS -evalue 1e-30 -qcov_h

[ok] Process 31058 (/bin/sh -c blastp -query - -db /home/anthonyw/anaconda3/db/kingdom/Bacteria/IS -evalue 1e-30 -qcov_h

[ok] Process 31061 (/bin/sh -c blastp -query - -db /home/anthonyw/anaconda3/db/kingdom/Bacteria/IS -evalue 1e-30 -qcov_h

[ok] Process 31064 (/bin/sh -c blastp -query - -db /home/anthonyw/anaconda3/db/kingdom/Bacteria/IS -evalue 1e-30 -qcov_h

[ok] Process 31069 (/bin/sh -c blastp -query - -db /home/anthonyw/anaconda3/db/kingdom/Bacteria/IS -evalue 1e-30 -qcov_h

[ok] Process 31078 (/bin/sh -c blastp -query - -db /home/anthonyw/anaconda3/db/kingdom/Bacteria/IS -evalue 1e-30 -qcov_h

[ok] Process 31113 (/bin/sh -c blastp -query - -db /home/anthonyw/anaconda3/db/kingdom/Bacteria/AMR -evalue 9.9999999999

[ok] Process 31115 (/bin/sh -c blastp -query - -db /home/anthonyw/anaconda3/db/kingdom/Bacteria/AMR -evalue 9.9999999999

[ok] Process 31119 (/bin/sh -c blastp -query - -db /home/anthonyw/anaconda3/db/kingdom/Bacteria/AMR -evalue 9.9999999999

[ok] Process 31130 (/bin/sh -c blastp -query - -db /home/anthonyw/anaconda3/db/kingdom/Bacteria/AMR -evalue 9.9999999999

[ok] Process 31123 (/bin/sh -c blastp -query - -db /home/anthonyw/anaconda3/db/kingdom/Bacteria/AMR -evalue 9.9999999999

[ok] Process 31125 (/bin/sh -c blastp -query - -db /home/anthonyw/anaconda3/db/kingdom/Bacteria/AMR -evalue 9.9999999999

[ok] Process 31128 (/bin/sh -c blastp -query - -db /home/anthonyw/anaconda3/db/kingdom/Bacteria/AMR -evalue 9.9999999999

[ok] Process 31136 (/bin/sh -c blastp -query - -db /home/anthonyw/anaconda3/db/kingdom/Bacteria/AMR -evalue 9.9999999999

[ok] Process 31148 (/bin/sh -c blastp -query - -db /home/anthonyw/anaconda3/db/kingdom/Bacteria/AMR -evalue 9.9999999999

[ok] Process 31156 (/bin/sh -c blastp -query - -db /home/anthonyw/anaconda3/db/kingdom/Bacteria/AMR -evalue 9.9999999999

[ok] Process 31159 (/bin/sh -c blastp -query - -db /home/anthonyw/anaconda3/db/kingdom/Bacteria/AMR -evalue 9.9999999999

[ok] Process 31168 (/bin/sh -c blastp -query - -db /home/anthonyw/anaconda3/db/kingdom/Bacteria/AMR -evalue 9.9999999999

[ok] Process 31173 (/bin/sh -c blastp -query - -db /home/anthonyw/anaconda3/db/kingdom/Bacteria/AMR -evalue 9.9999999999

[ok] Process 31176 (/bin/sh -c blastp -query - -db /home/anthonyw/anaconda3/db/kingdom/Bacteria/AMR -evalue 9.9999999999

[ok] Process 31181 (/bin/sh -c blastp -query - -db /home/anthonyw/anaconda3/db/kingdom/Bacteria/AMR -evalue 9.9999999999

[ok] Process 31184 (/bin/sh -c blastp -query - -db /home/anthonyw/anaconda3/db/kingdom/Bacteria/AMR -evalue 9.9999999999

[ok] Process 31192 (/bin/sh -c blastp -query - -db /home/anthonyw/anaconda3/db/kingdom/Bacteria/AMR -evalue 9.9999999999

[ok] Process 31227 (/bin/sh -c blastp -query - -db /home/anthonyw/anaconda3/db/kingdom/Bacteria/sprot -evalue 1e-09 -qco

[ok] Process 31229 (/bin/sh -c blastp -query - -db /home/anthonyw/anaconda3/db/kingdom/Bacteria/sprot -evalue 1e-09 -qco

[ok] Process 31232 (/bin/sh -c blastp -query - -db /home/anthonyw/anaconda3/db/kingdom/Bacteria/sprot -evalue 1e-09 -qco

[ok] Process 31237 (/bin/sh -c blastp -query - -db /home/anthonyw/anaconda3/db/kingdom/Bacteria/sprot -evalue 1e-09 -qco

[ok] Process 31246 (/bin/sh -c blastp -query - -db /home/anthonyw/anaconda3/db/kingdom/Bacteria/sprot -evalue 1e-09 -qco

[ok] Process 31239 (/bin/sh -c blastp -query - -db /home/anthonyw/anaconda3/db/kingdom/Bacteria/sprot -evalue 1e-09 -qco

[ok] Process 31241 (/bin/sh -c blastp -query - -db /home/anthonyw/anaconda3/db/kingdom/Bacteria/sprot -evalue 1e-09 -qco

[ok] Process 31250 (/bin/sh -c blastp -query - -db /home/anthonyw/anaconda3/db/kingdom/Bacteria/sprot -evalue 1e-09 -qco

[ok] Process 31273 (/bin/sh -c blastp -query - -db /home/anthonyw/anaconda3/db/kingdom/Bacteria/sprot -evalue 1e-09 -qco

[ok] Process 31278 (/bin/sh -c blastp -query - -db /home/anthonyw/anaconda3/db/kingdom/Bacteria/sprot -evalue 1e-09 -qco

[ok] Process 31286 (/bin/sh -c blastp -query - -db /home/anthonyw/anaconda3/db/kingdom/Bacteria/sprot -evalue 1e-09 -qco

[ok] Process 31293 (/bin/sh -c blastp -query - -db /home/anthonyw/anaconda3/db/kingdom/Bacteria/sprot -evalue 1e-09 -qco

[ok] Process 31298 (/bin/sh -c blastp -query - -db /home/anthonyw/anaconda3/db/kingdom/Bacteria/sprot -evalue 1e-09 -qco

[ok] Process 31305 (/bin/sh -c blastp -query - -db /home/anthonyw/anaconda3/db/kingdom/Bacteria/sprot -evalue 1e-09 -qco

[ok] Process 31308 (/bin/sh -c blastp -query - -db /home/anthonyw/anaconda3/db/kingdom/Bacteria/sprot -evalue 1e-09 -qco

[ok] Process 31310 (/bin/sh -c blastp -query - -db /home/anthonyw/anaconda3/db/kingdom/Bacteria/sprot -evalue 1e-09 -qco

[ok] Process 31335 (/bin/sh -c blastp -query - -db /home/anthonyw/anaconda3/db/kingdom/Bacteria/sprot -evalue 1e-09 -qco

[ok] Process 31370 (/bin/sh -c hmmscan --noali --notextw --acc -E 1e-09 --cpu 1 /home/anthonyw/anaconda3/db/hmm/HAMAP.hm

[ok] Process 31372 (/bin/sh -c hmmscan --noali --notextw --acc -E 1e-09 --cpu 1 /home/anthonyw/anaconda3/db/hmm/HAMAP.hm

[ok] Process 31376 (/bin/sh -c hmmscan --noali --notextw --acc -E 1e-09 --cpu 1 /home/anthonyw/anaconda3/db/hmm/HAMAP.hm

[ok] Process 31378 (/bin/sh -c hmmscan --noali --notextw --acc -E 1e-09 --cpu 1 /home/anthonyw/anaconda3/db/hmm/HAMAP.hm

[ok] Process 31381 (/bin/sh -c hmmscan --noali --notextw --acc -E 1e-09 --cpu 1 /home/anthonyw/anaconda3/db/hmm/HAMAP.hm

[ok] Process 31383 (/bin/sh -c hmmscan --noali --notextw --acc -E 1e-09 --cpu 1 /home/anthonyw/anaconda3/db/hmm/HAMAP.hm

[ok] Process 31385 (/bin/sh -c hmmscan --noali --notextw --acc -E 1e-09 --cpu 1 /home/anthonyw/anaconda3/db/hmm/HAMAP.hm

[ok] Process 31402 (/bin/sh -c hmmscan --noali --notextw --acc -E 1e-09 --cpu 1 /home/anthonyw/anaconda3/db/hmm/HAMAP.hm

[ok] Process 31949 (/bin/sh -c hmmscan --noali --notextw --acc -E 1e-09 --cpu 1 /home/anthonyw/anaconda3/db/hmm/HAMAP.hm

[ok] Process 31953 (/bin/sh -c hmmscan --noali --notextw --acc -E 1e-09 --cpu 1 /home/anthonyw/anaconda3/db/hmm/HAMAP.hm

[ok] Process 31969 (/bin/sh -c hmmscan --noali --notextw --acc -E 1e-09 --cpu 1 /home/anthonyw/anaconda3/db/hmm/HAMAP.hm

[ok] Process 32058 (/bin/sh -c hmmscan --noali --notextw --acc -E 1e-09 --cpu 1 /home/anthonyw/anaconda3/db/hmm/HAMAP.hm

[ok] Process 32100 (/bin/sh -c hmmscan --noali --notextw --acc -E 1e-09 --cpu 1 /home/anthonyw/anaconda3/db/hmm/HAMAP.hm

[ok] Process 32129 (/bin/sh -c hmmscan --noali --notextw --acc -E 1e-09 --cpu 1 /home/anthonyw/anaconda3/db/hmm/HAMAP.hm

[ok] Process 32169 (/bin/sh -c hmmscan --noali --notextw --acc -E 1e-09 --cpu 1 /home/anthonyw/anaconda3/db/hmm/HAMAP.hm

[ok] Process 32186 (/bin/sh -c hmmscan --noali --notextw --acc -E 1e-09 --cpu 1 /home/anthonyw/anaconda3/db/hmm/HAMAP.hm

[ok] Process 32370 (/bin/sh -c hmmscan --noali --notextw --acc -E 1e-09 --cpu 1 /home/anthonyw/anaconda3/db/hmm/HAMAP.hm

[ok] Process 31003 (blastp -query - -db /home/anthonyw/anaconda3/db/kingdom/Bacteria/IS -evalue 1e-30 -qcov_hsp_perc 90

[ok] Process 31006 (blastp -query - -db /home/anthonyw/anaconda3/db/kingdom/Bacteria/IS -evalue 1e-30 -qcov_hsp_perc 90

[ok] Process 31005 (blastp -query - -db /home/anthonyw/anaconda3/db/kingdom/Bacteria/IS -evalue 1e-30 -qcov_hsp_perc 90

[ok] Process 31013 (blastp -query - -db /home/anthonyw/anaconda3/db/kingdom/Bacteria/IS -evalue 1e-30 -qcov_hsp_perc 90

[ok] Process 31020 (blastp -query - -db /home/anthonyw/anaconda3/db/kingdom/Bacteria/IS -evalue 1e-30 -qcov_hsp_perc 90

[ok] Process 31016 (blastp -query - -db /home/anthonyw/anaconda3/db/kingdom/Bacteria/IS -evalue 1e-30 -qcov_hsp_perc 90

[ok] Process 31017 (blastp -query - -db /home/anthonyw/anaconda3/db/kingdom/Bacteria/IS -evalue 1e-30 -qcov_hsp_perc 90

[ok] Process 31022 (blastp -query - -db /home/anthonyw/anaconda3/db/kingdom/Bacteria/IS -evalue 1e-30 -qcov_hsp_perc 90

[ok] Process 31035 (blastp -query - -db /home/anthonyw/anaconda3/db/kingdom/Bacteria/IS -evalue 1e-30 -qcov_hsp_perc 90

[ok] Process 31042 (blastp -query - -db /home/anthonyw/anaconda3/db/kingdom/Bacteria/IS -evalue 1e-30 -qcov_hsp_perc 90

[ok] Process 31047 (blastp -query - -db /home/anthonyw/anaconda3/db/kingdom/Bacteria/IS -evalue 1e-30 -qcov_hsp_perc 90

[ok] Process 31054 (blastp -query - -db /home/anthonyw/anaconda3/db/kingdom/Bacteria/IS -evalue 1e-30 -qcov_hsp_perc 90

[ok] Process 31059 (blastp -query - -db /home/anthonyw/anaconda3/db/kingdom/Bacteria/IS -evalue 1e-30 -qcov_hsp_perc 90

[ok] Process 31062 (blastp -query - -db /home/anthonyw/anaconda3/db/kingdom/Bacteria/IS -evalue 1e-30 -qcov_hsp_perc 90

[ok] Process 31065 (blastp -query - -db /home/anthonyw/anaconda3/db/kingdom/Bacteria/IS -evalue 1e-30 -qcov_hsp_perc 90

[ok] Process 31070 (blastp -query - -db /home/anthonyw/anaconda3/db/kingdom/Bacteria/IS -evalue 1e-30 -qcov_hsp_perc 90

[ok] Process 31079 (blastp -query - -db /home/anthonyw/anaconda3/db/kingdom/Bacteria/IS -evalue 1e-30 -qcov_hsp_perc 90

[ok] Process 31116 (blastp -query - -db /home/anthonyw/anaconda3/db/kingdom/Bacteria/AMR -evalue 9.99999999999999e-301 -

[ok] Process 31118 (blastp -query - -db /home/anthonyw/anaconda3/db/kingdom/Bacteria/AMR -evalue 9.99999999999999e-301 -

[ok] Process 31121 (blastp -query - -db /home/anthonyw/anaconda3/db/kingdom/Bacteria/AMR -evalue 9.99999999999999e-301 -

[ok] Process 31132 (blastp -query - -db /home/anthonyw/anaconda3/db/kingdom/Bacteria/AMR -evalue 9.99999999999999e-301 -

[ok] Process 31129 (blastp -query - -db /home/anthonyw/anaconda3/db/kingdom/Bacteria/AMR -evalue 9.99999999999999e-301 -

[ok] Process 31133 (blastp -query - -db /home/anthonyw/anaconda3/db/kingdom/Bacteria/AMR -evalue 9.99999999999999e-301 -

[ok] Process 31131 (blastp -query - -db /home/anthonyw/anaconda3/db/kingdom/Bacteria/AMR -evalue 9.99999999999999e-301 -

[ok] Process 31137 (blastp -query - -db /home/anthonyw/anaconda3/db/kingdom/Bacteria/AMR -evalue 9.99999999999999e-301 -

[ok] Process 31149 (blastp -query - -db /home/anthonyw/anaconda3/db/kingdom/Bacteria/AMR -evalue 9.99999999999999e-301 -

[ok] Process 31157 (blastp -query - -db /home/anthonyw/anaconda3/db/kingdom/Bacteria/AMR -evalue 9.99999999999999e-301 -

[ok] Process 31160 (blastp -query - -db /home/anthonyw/anaconda3/db/kingdom/Bacteria/AMR -evalue 9.99999999999999e-301 -

[ok] Process 31169 (blastp -query - -db /home/anthonyw/anaconda3/db/kingdom/Bacteria/AMR -evalue 9.99999999999999e-301 -

[ok] Process 31174 (blastp -query - -db /home/anthonyw/anaconda3/db/kingdom/Bacteria/AMR -evalue 9.99999999999999e-301 -

[ok] Process 31177 (blastp -query - -db /home/anthonyw/anaconda3/db/kingdom/Bacteria/AMR -evalue 9.99999999999999e-301 -

[ok] Process 31182 (blastp -query - -db /home/anthonyw/anaconda3/db/kingdom/Bacteria/AMR -evalue 9.99999999999999e-301 -

[ok] Process 31185 (blastp -query - -db /home/anthonyw/anaconda3/db/kingdom/Bacteria/AMR -evalue 9.99999999999999e-301 -

[ok] Process 31193 (blastp -query - -db /home/anthonyw/anaconda3/db/kingdom/Bacteria/AMR -evalue 9.99999999999999e-301 -

[ok] Process 31230 (blastp -query - -db /home/anthonyw/anaconda3/db/kingdom/Bacteria/sprot -evalue 1e-09 -qcov_hsp_perc

[ok] Process 31233 (blastp -query - -db /home/anthonyw/anaconda3/db/kingdom/Bacteria/sprot -evalue 1e-09 -qcov_hsp_perc

[ok] Process 31236 (blastp -query - -db /home/anthonyw/anaconda3/db/kingdom/Bacteria/sprot -evalue 1e-09 -qcov_hsp_perc

[ok] Process 31245 (blastp -query - -db /home/anthonyw/anaconda3/db/kingdom/Bacteria/sprot -evalue 1e-09 -qcov_hsp_perc

[ok] Process 31247 (blastp -query - -db /home/anthonyw/anaconda3/db/kingdom/Bacteria/sprot -evalue 1e-09 -qcov_hsp_perc

[ok] Process 31243 (blastp -query - -db /home/anthonyw/anaconda3/db/kingdom/Bacteria/sprot -evalue 1e-09 -qcov_hsp_perc

[ok] Process 31244 (blastp -query - -db /home/anthonyw/anaconda3/db/kingdom/Bacteria/sprot -evalue 1e-09 -qcov_hsp_perc

[ok] Process 31251 (blastp -query - -db /home/anthonyw/anaconda3/db/kingdom/Bacteria/sprot -evalue 1e-09 -qcov_hsp_perc

[ok] Process 31274 (blastp -query - -db /home/anthonyw/anaconda3/db/kingdom/Bacteria/sprot -evalue 1e-09 -qcov_hsp_perc

[ok] Process 31279 (blastp -query - -db /home/anthonyw/anaconda3/db/kingdom/Bacteria/sprot -evalue 1e-09 -qcov_hsp_perc

[ok] Process 31287 (blastp -query - -db /home/anthonyw/anaconda3/db/kingdom/Bacteria/sprot -evalue 1e-09 -qcov_hsp_perc

[ok] Process 31294 (blastp -query - -db /home/anthonyw/anaconda3/db/kingdom/Bacteria/sprot -evalue 1e-09 -qcov_hsp_perc

[ok] Process 31299 (blastp -query - -db /home/anthonyw/anaconda3/db/kingdom/Bacteria/sprot -evalue 1e-09 -qcov_hsp_perc

[ok] Process 31306 (blastp -query - -db /home/anthonyw/anaconda3/db/kingdom/Bacteria/sprot -evalue 1e-09 -qcov_hsp_perc

[ok] Process 31311 (blastp -query - -db /home/anthonyw/anaconda3/db/kingdom/Bacteria/sprot -evalue 1e-09 -qcov_hsp_perc

[ok] Process 31312 (blastp -query - -db /home/anthonyw/anaconda3/db/kingdom/Bacteria/sprot -evalue 1e-09 -qcov_hsp_perc

[ok] Process 31336 (blastp -query - -db /home/anthonyw/anaconda3/db/kingdom/Bacteria/sprot -evalue 1e-09 -qcov_hsp_perc

[ok] Process 31374 (hmmscan --noali --notextw --acc -E 1e-09 --cpu 1 /home/anthonyw/anaconda3/db/hmm/HAMAP.hmm /dev/stdi

[ok] Process 31379 (hmmscan --noali --notextw --acc -E 1e-09 --cpu 1 /home/anthonyw/anaconda3/db/hmm/HAMAP.hmm /dev/stdi

[ok] Process 31387 (hmmscan --noali --notextw --acc -E 1e-09 --cpu 1 /home/anthonyw/anaconda3/db/hmm/HAMAP.hmm /dev/stdi

[ok] Process 31396 (hmmscan --noali --notextw --acc -E 1e-09 --cpu 1 /home/anthonyw/anaconda3/db/hmm/HAMAP.hmm /dev/stdi

[ok] Process 31390 (hmmscan --noali --notextw --acc -E 1e-09 --cpu 1 /home/anthonyw/anaconda3/db/hmm/HAMAP.hmm /dev/stdi

[ok] Process 31389 (hmmscan --noali --notextw --acc -E 1e-09 --cpu 1 /home/anthonyw/anaconda3/db/hmm/HAMAP.hmm /dev/stdi

[ok] Process 31393 (hmmscan --noali --notextw --acc -E 1e-09 --cpu 1 /home/anthonyw/anaconda3/db/hmm/HAMAP.hmm /dev/stdi

[ok] Process 31403 (hmmscan --noali --notextw --acc -E 1e-09 --cpu 1 /home/anthonyw/anaconda3/db/hmm/HAMAP.hmm /dev/stdi

[ok] Process 31950 (hmmscan --noali --notextw --acc -E 1e-09 --cpu 1 /home/anthonyw/anaconda3/db/hmm/HAMAP.hmm /dev/stdi

[ok] Process 31954 (hmmscan --noali --notextw --acc -E 1e-09 --cpu 1 /home/anthonyw/anaconda3/db/hmm/HAMAP.hmm /dev/stdi

[ok] Process 31970 (hmmscan --noali --notextw --acc -E 1e-09 --cpu 1 /home/anthonyw/anaconda3/db/hmm/HAMAP.hmm /dev/stdi

[ok] Process 32059 (hmmscan --noali --notextw --acc -E 1e-09 --cpu 1 /home/anthonyw/anaconda3/db/hmm/HAMAP.hmm /dev/stdi

[ok] Process 32101 (hmmscan --noali --notextw --acc -E 1e-09 --cpu 1 /home/anthonyw/anaconda3/db/hmm/HAMAP.hmm /dev/stdi

[ok] Process 32130 (hmmscan --noali --notextw --acc -E 1e-09 --cpu 1 /home/anthonyw/anaconda3/db/hmm/HAMAP.hmm /dev/stdi

[ok] Process 32170 (hmmscan --noali --notextw --acc -E 1e-09 --cpu 1 /home/anthonyw/anaconda3/db/hmm/HAMAP.hmm /dev/stdi

[ok] Process 32187 (hmmscan --noali --notextw --acc -E 1e-09 --cpu 1 /home/anthonyw/anaconda3/db/hmm/HAMAP.hmm /dev/stdi

[ok] Process 32373 (hmmscan --noali --notextw --acc -E 1e-09 --cpu 1 /home/anthonyw/anaconda3/db/hmm/HAMAP.hmm /dev/stdi

[info] Verification complete

# Genome annotation verification – EnvDst2 (warnings)

[ok] Process 15985 (/bin/sh -c hmmscan --noali --notextw --acc -E 1e-09 --cpu 1 /home/anthonyw/anaconda3/db/hmm/HAMAP.hm

[ok] Process 16047 (/bin/sh -c hmmscan --noali --notextw --acc -E 1e-09 --cpu 1 /home/anthonyw/anaconda3/db/hmm/HAMAP.hm

[ok] Process 16164 (/bin/sh -c hmmscan --noali --notextw --acc -E 1e-09 --cpu 1 /home/anthonyw/anaconda3/db/hmm/HAMAP.hm

[ok] Process 14773 (blastp -query - -db /home/anthonyw/anaconda3/db/kingdom/Bacteria/IS -evalue 1e-30 -qcov_hsp_perc 90

[warning] Process 14773 (blastp -query - -db /home/anthonyw/anaconda3/db/kingdom/Bacteria/IS -evalue 1e-30 -qcov_hsp_per

[ok] Process 14776 (blastp -query - -db /home/anthonyw/anaconda3/db/kingdom/Bacteria/IS -evalue 1e-30 -qcov_hsp_perc 90

[warning] Process 14776 (blastp -query - -db /home/anthonyw/anaconda3/db/kingdom/Bacteria/IS -evalue 1e-30 -qcov_hsp_per

[ok] Process 14779 (blastp -query - -db /home/anthonyw/anaconda3/db/kingdom/Bacteria/IS -evalue 1e-30 -qcov_hsp_perc 90

[warning] Process 14779 (blastp -query - -db /home/anthonyw/anaconda3/db/kingdom/Bacteria/IS -evalue 1e-30 -qcov_hsp_per

[ok] Process 14783 (blastp -query - -db /home/anthonyw/anaconda3/db/kingdom/Bacteria/IS -evalue 1e-30 -qcov_hsp_perc 90

[warning] Process 14783 (blastp -query - -db /home/anthonyw/anaconda3/db/kingdom/Bacteria/IS -evalue 1e-30 -qcov_hsp_per

[ok] Process 14784 (blastp -query - -db /home/anthonyw/anaconda3/db/kingdom/Bacteria/IS -evalue 1e-30 -qcov_hsp_perc 90

[warning] Process 14784 (blastp -query - -db /home/anthonyw/anaconda3/db/kingdom/Bacteria/IS -evalue 1e-30 -qcov_hsp_per

[ok] Process 14785 (blastp -query - -db /home/anthonyw/anaconda3/db/kingdom/Bacteria/IS -evalue 1e-30 -qcov_hsp_perc 90

[warning] Process 14785 (blastp -query - -db /home/anthonyw/anaconda3/db/kingdom/Bacteria/IS -evalue 1e-30 -qcov_hsp_per

[ok] Process 14791 (blastp -query - -db /home/anthonyw/anaconda3/db/kingdom/Bacteria/IS -evalue 1e-30 -qcov_hsp_perc 90

[warning] Process 14791 (blastp -query - -db /home/anthonyw/anaconda3/db/kingdom/Bacteria/IS -evalue 1e-30 -qcov_hsp_per

[ok] Process 14796 (blastp -query - -db /home/anthonyw/anaconda3/db/kingdom/Bacteria/IS -evalue 1e-30 -qcov_hsp_perc 90

[warning] Process 14796 (blastp -query - -db /home/anthonyw/anaconda3/db/kingdom/Bacteria/IS -evalue 1e-30 -qcov_hsp_per

[ok] Process 14804 (blastp -query - -db /home/anthonyw/anaconda3/db/kingdom/Bacteria/IS -evalue 1e-30 -qcov_hsp_perc 90

[warning] Process 14804 (blastp -query - -db /home/anthonyw/anaconda3/db/kingdom/Bacteria/IS -evalue 1e-30 -qcov_hsp_per

[ok] Process 14813 (blastp -query - -db /home/anthonyw/anaconda3/db/kingdom/Bacteria/IS -evalue 1e-30 -qcov_hsp_perc 90

[warning] Process 14813 (blastp -query - -db /home/anthonyw/anaconda3/db/kingdom/Bacteria/IS -evalue 1e-30 -qcov_hsp_per

[ok] Process 14816 (blastp -query - -db /home/anthonyw/anaconda3/db/kingdom/Bacteria/IS -evalue 1e-30 -qcov_hsp_perc 90

[warning] Process 14816 (blastp -query - -db /home/anthonyw/anaconda3/db/kingdom/Bacteria/IS -evalue 1e-30 -qcov_hsp_per

[ok] Process 14829 (blastp -query - -db /home/anthonyw/anaconda3/db/kingdom/Bacteria/IS -evalue 1e-30 -qcov_hsp_perc 90

[warning] Process 14829 (blastp -query - -db /home/anthonyw/anaconda3/db/kingdom/Bacteria/IS -evalue 1e-30 -qcov_hsp_per

[ok] Process 14833 (blastp -query - -db /home/anthonyw/anaconda3/db/kingdom/Bacteria/IS -evalue 1e-30 -qcov_hsp_perc 90

[warning] Process 14833 (blastp -query - -db /home/anthonyw/anaconda3/db/kingdom/Bacteria/IS -evalue 1e-30 -qcov_hsp_per

[ok] Process 14831 (blastp -query - -db /home/anthonyw/anaconda3/db/kingdom/Bacteria/IS -evalue 1e-30 -qcov_hsp_perc 90

[warning] Process 14831 (blastp -query - -db /home/anthonyw/anaconda3/db/kingdom/Bacteria/IS -evalue 1e-30 -qcov_hsp_per

[ok] Process 14836 (blastp -query - -db /home/anthonyw/anaconda3/db/kingdom/Bacteria/IS -evalue 1e-30 -qcov_hsp_perc 90

[warning] Process 14836 (blastp -query - -db /home/anthonyw/anaconda3/db/kingdom/Bacteria/IS -evalue 1e-30 -qcov_hsp_per

[ok] Process 14844 (blastp -query - -db /home/anthonyw/anaconda3/db/kingdom/Bacteria/IS -evalue 1e-30 -qcov_hsp_perc 90

[warning] Process 14844 (blastp -query - -db /home/anthonyw/anaconda3/db/kingdom/Bacteria/IS -evalue 1e-30 -qcov_hsp_per

[ok] Process 14852 (blastp -query - -db /home/anthonyw/anaconda3/db/kingdom/Bacteria/IS -evalue 1e-30 -qcov_hsp_perc 90

[warning] Process 14852 (blastp -query - -db /home/anthonyw/anaconda3/db/kingdom/Bacteria/IS -evalue 1e-30 -qcov_hsp_per

[ok] Process 14890 (blastp -query - -db /home/anthonyw/anaconda3/db/kingdom/Bacteria/AMR -evalue 9.99999999999999e-301 –

[warning] Process 14890 (blastp -query - -db /home/anthonyw/anaconda3/db/kingdom/Bacteria/AMR -evalue 9.99999999[0/8042]

[ok] Process 14895 (blastp -query - -db /home/anthonyw/anaconda3/db/kingdom/Bacteria/AMR -evalue 9.99999999999999e-301 -

[warning] Process 14895 (blastp -query - -db /home/anthonyw/anaconda3/db/kingdom/Bacteria/AMR -evalue 9.99999999999999e-

[ok] Process 14898 (blastp -query - -db /home/anthonyw/anaconda3/db/kingdom/Bacteria/AMR -evalue 9.99999999999999e-301 -

[warning] Process 14898 (blastp -query - -db /home/anthonyw/anaconda3/db/kingdom/Bacteria/AMR -evalue 9.99999999999999e-

[ok] Process 14900 (blastp -query - -db /home/anthonyw/anaconda3/db/kingdom/Bacteria/AMR -evalue 9.99999999999999e-301 -

[warning] Process 14900 (blastp -query - -db /home/anthonyw/anaconda3/db/kingdom/Bacteria/AMR -evalue 9.99999999999999e-

[ok] Process 14906 (blastp -query - -db /home/anthonyw/anaconda3/db/kingdom/Bacteria/AMR -evalue 9.99999999999999e-301 -

[warning] Process 14906 (blastp -query - -db /home/anthonyw/anaconda3/db/kingdom/Bacteria/AMR -evalue 9.99999999999999e-

[ok] Process 14909 (blastp -query - -db /home/anthonyw/anaconda3/db/kingdom/Bacteria/AMR -evalue 9.99999999999999e-301 -

[warning] Process 14909 (blastp -query - -db /home/anthonyw/anaconda3/db/kingdom/Bacteria/AMR -evalue 9.99999999999999e-

[ok] Process 14907 (blastp -query - -db /home/anthonyw/anaconda3/db/kingdom/Bacteria/AMR -evalue 9.99999999999999e-301 -

[warning] Process 14907 (blastp -query - -db /home/anthonyw/anaconda3/db/kingdom/Bacteria/AMR -evalue 9.99999999999999e-

[ok] Process 14913 (blastp -query - -db /home/anthonyw/anaconda3/db/kingdom/Bacteria/AMR -evalue 9.99999999999999e-301 -

[warning] Process 14913 (blastp -query - -db /home/anthonyw/anaconda3/db/kingdom/Bacteria/AMR -evalue 9.99999999999999e-

[ok] Process 14921 (blastp -query - -db /home/anthonyw/anaconda3/db/kingdom/Bacteria/AMR -evalue 9.99999999999999e-301 -

[warning] Process 14921 (blastp -query - -db /home/anthonyw/anaconda3/db/kingdom/Bacteria/AMR -evalue 9.99999999999999e-

[ok] Process 14929 (blastp -query - -db /home/anthonyw/anaconda3/db/kingdom/Bacteria/AMR -evalue 9.99999999999999e-301 -

[warning] Process 14929 (blastp -query - -db /home/anthonyw/anaconda3/db/kingdom/Bacteria/AMR -evalue 9.99999999999999e-

[ok] Process 14933 (blastp -query - -db /home/anthonyw/anaconda3/db/kingdom/Bacteria/AMR -evalue 9.99999999999999e-301 -

[warning] Process 14933 (blastp -query - -db /home/anthonyw/anaconda3/db/kingdom/Bacteria/AMR -evalue 9.99999999999999e-

[ok] Process 14936 (blastp -query - -db /home/anthonyw/anaconda3/db/kingdom/Bacteria/AMR -evalue 9.99999999999999e-301 -

[warning] Process 14936 (blastp -query - -db /home/anthonyw/anaconda3/db/kingdom/Bacteria/AMR -evalue 9.99999999999999e-

[ok] Process 14947 (blastp -query - -db /home/anthonyw/anaconda3/db/kingdom/Bacteria/AMR -evalue 9.99999999999999e-301 -

[warning] Process 14947 (blastp -query - -db /home/anthonyw/anaconda3/db/kingdom/Bacteria/AMR -evalue 9.99999999999999e-

[ok] Process 14955 (blastp -query - -db /home/anthonyw/anaconda3/db/kingdom/Bacteria/AMR -evalue 9.99999999999999e-301 -

[warning] Process 14955 (blastp -query - -db /home/anthonyw/anaconda3/db/kingdom/Bacteria/AMR -evalue 9.99999999999999e-

[ok] Process 14954 (blastp -query - -db /home/anthonyw/anaconda3/db/kingdom/Bacteria/AMR -evalue 9.99999999999999e-301 -

[warning] Process 14954 (blastp -query - -db /home/anthonyw/anaconda3/db/kingdom/Bacteria/AMR -evalue 9.99999999999999e-

[ok] Process 14956 (blastp -query - -db /home/anthonyw/anaconda3/db/kingdom/Bacteria/AMR -evalue 9.99999999999999e-301 -

[warning] Process 14956 (blastp -query - -db /home/anthonyw/anaconda3/db/kingdom/Bacteria/AMR -evalue 9.99999999999999e-

[ok] Process 14968 (blastp -query - -db /home/anthonyw/anaconda3/db/kingdom/Bacteria/AMR -evalue 9.99999999999999e-301 -

[warning] Process 14968 (blastp -query - -db /home/anthonyw/anaconda3/db/kingdom/Bacteria/AMR -evalue 9.99999999999999e-

[ok] Process 15005 (blastp -query - -db /home/anthonyw/anaconda3/db/kingdom/Bacteria/sprot -evalue 1e-09 -qcov_hsp_perc

[warning] Process 15005 (blastp -query - -db /home/anthonyw/anaconda3/db/kingdom/Bacteria/sprot -evalue 1e-09 -qcov_hsp_

[ok] Process 15009 (blastp -query - -db /home/anthonyw/anaconda3/db/kingdom/Bacteria/sprot -evalue 1e-09 -qcov_hsp_perc

[warning] Process 15009 (blastp -query - -db /home/anthonyw/anaconda3/db/kingdom/Bacteria/sprot -evalue 1e-09 -qcov_hsp_

[ok] Process 15013 (blastp -query - -db /home/anthonyw/anaconda3/db/kingdom/Bacteria/sprot -evalue 1e-09 -qcov_hsp_perc

[warning] Process 15013 (blastp -query - -db /home/anthonyw/anaconda3/db/kingdom/Bacteria/sprot -evalue 1e-09 -qcov_hsp_

[ok] Process 15018 (blastp -query - -db /home/anthonyw/anaconda3/db/kingdom/Bacteria/sprot -evalue 1e-09 -qcov_hsp_perc

[warning] Process 15018 (blastp -query - -db /home/anthonyw/anaconda3/db/kingdom/Bacteria/sprot -evalue 1e-09 -qcov_hsp_

[ok] Process 15017 (blastp -query - -db /home/anthonyw/anaconda3/db/kingdom/Bacteria/sprot -evalue 1e-09 -qcov_hsp_perc

[warning] Process 15017 (blastp -query - -db /home/anthonyw/anaconda3/db/kingdom/Bacteria/sprot -evalue 1e-09 -qcov_hsp_

[ok] Process 15022 (blastp -query - -db /home/anthonyw/anaconda3/db/kingdom/Bacteria/sprot -evalue 1e-09 -qcov_hsp_perc

[warning] Process 15022 (blastp -query - -db /home/anthonyw/anaconda3/db/kingdom/Bacteria/sprot -evalue 1e-09 -qcov_hsp_

[ok] Process 15023 (blastp -query - -db /home/anthonyw/anaconda3/db/kingdom/Bacteria/sprot -evalue 1e-09 -qcov_hsp_perc

[warning] Process 15023 (blastp -query - -db /home/anthonyw/anaconda3/db/kingdom/Bacteria/sprot -evalue 1e-09 -qcov_hsp_

[ok] Process 15027 (blastp -query - -db /home/anthonyw/anaconda3/db/kingdom/Bacteria/sprot -evalue 1e-09 -qcov_hsp_perc

[warning] Process 15027 (blastp -query - -db /home/anthonyw/anaconda3/db/kingdom/Bacteria/sprot -evalue 1e-09 -qcov_hsp_

[ok] Process 15056 (blastp -query - -db /home/anthonyw/anaconda3/db/kingdom/Bacteria/sprot -evalue 1e-09 -qcov_hsp_perc

[warning] Process 15056 (blastp -query - -db /home/anthonyw/anaconda3/db/kingdom/Bacteria/sprot -evalue 1e-09 -qcov_hsp_

[ok] Process 15061 (blastp -query - -db /home/anthonyw/anaconda3/db/kingdom/Bacteria/sprot -evalue 1e-09 -qcov_hsp_perc

[warning] Process 15061 (blastp -query - -db /home/anthonyw/anaconda3/db/kingdom/Bacteria/sprot -evalue 1e-09 -qcov_hsp_

[ok] Process 15072 (blastp -query - -db /home/anthonyw/anaconda3/db/kingdom/Bacteria/sprot -evalue 1e-09 -qcov_hsp_perc

[warning] Process 15072 (blastp -query - -db /home/anthonyw/anaconda3/db/kingdom/Bacteria/sprot -evalue 1e-09 -qcov_hsp_

[ok] Process 15071 (blastp -query - -db /home/anthonyw/anaconda3/db/kingdom/Bacteria/sprot -evalue 1e-09 -qcov_hsp_perc

[warning] Process 15071 (blastp -query - -db /home/anthonyw/anaconda3/db/kingdom/Bacteria/sprot -evalue 1e-09 -qcov_hsp_

[ok] Process 15079 (blastp -query - -db /home/anthonyw/anaconda3/db/kingdom/Bacteria/sprot -evalue 1e-09 -qcov_hsp_perc

[warning] Process 15079 (blastp -query - -db /home/anthonyw/anaconda3/db/kingdom/Bacteria/sprot -evalue 1e-09 -qcov_hsp_

[ok] Process 15082 (blastp -query - -db /home/anthonyw/anaconda3/db/kingdom/Bacteria/sprot -evalue 1e-09 -qcov_hsp_perc

[warning] Process 15082 (blastp -query - -db /home/anthonyw/anaconda3/db/kingdom/Bacteria/sprot -evalue 1e-09 -qcov_hsp_

[ok] Process 15091 (blastp -query - -db /home/anthonyw/anaconda3/db/kingdom/Bacteria/sprot -evalue 1e-09 -qcov_hsp_perc

[warning] Process 15091 (blastp -query - -db /home/anthonyw/anaconda3/db/kingdom/Bacteria/sprot -evalue 1e-09 -qcov_hsp_

[ok] Process 15094 (blastp -query - -db /home/anthonyw/anaconda3/db/kingdom/Bacteria/sprot -evalue 1e-09 -qcov_hsp_perc

[warning] Process 15094 (blastp -query - -db /home/anthonyw/anaconda3/db/kingdom/Bacteria/sprot -evalue 1e-09 -qcov_hsp_

[ok] Process 15117 (blastp -query - -db /home/anthonyw/anaconda3/db/kingdom/Bacteria/sprot -evalue 1e-09 -qcov_hsp_perc

[warning] Process 15117 (blastp -query - -db /home/anthonyw/anaconda3/db/kingdom/Bacteria/sprot -evalue 1e-09 -qcov_hsp_

[ok] Process 15155 (hmmscan --noali --notextw --acc -E 1e-09 --cpu 1 /home/anthonyw/anaconda3/db/hmm/HAMAP.hmm /dev/stdi

[ok] Process 15174 (hmmscan --noali --notextw --acc -E 1e-09 --cpu 1 /home/anthonyw/anaconda3/db/hmm/HAMAP.hmm /dev/stdi

[ok] Process 15160 (hmmscan --noali --notextw --acc -E 1e-09 --cpu 1 /home/anthonyw/anaconda3/db/hmm/HAMAP.hmm /dev/stdi

[ok] Process 15163 (hmmscan --noali --notextw --acc -E 1e-09 --cpu 1 /home/anthonyw/anaconda3/db/hmm/HAMAP.hmm /dev/stdi

[ok] Process 15169 (hmmscan --noali --notextw --acc -E 1e-09 --cpu 1 /home/anthonyw/anaconda3/db/hmm/HAMAP.hmm /dev/stdi

[ok] Process 15175 (hmmscan --noali --notextw --acc -E 1e-09 --cpu 1 /home/anthonyw/anaconda3/db/hmm/HAMAP.hmm /dev/stdi

[ok] Process 15172 (hmmscan --noali --notextw --acc -E 1e-09 --cpu 1 /home/anthonyw/anaconda3/db/hmm/HAMAP.hmm /dev/stdi

[ok] Process 15183 (hmmscan --noali --notextw --acc -E 1e-09 --cpu 1 /home/anthonyw/anaconda3/db/hmm/HAMAP.hmm /dev/stdi

[ok] Process 15725 (hmmscan --noali --notextw --acc -E 1e-09 --cpu 1 /home/anthonyw/anaconda3/db/hmm/HAMAP.hmm /dev/stdi

[ok] Process 15738 (hmmscan --noali --notextw --acc -E 1e-09 --cpu 1 /home/anthonyw/anaconda3/db/hmm/HAMAP.hmm /dev/stdi

[ok] Process 15767 (hmmscan --noali --notextw --acc -E 1e-09 --cpu 1 /home/anthonyw/anaconda3/db/hmm/HAMAP.hmm /dev/stdi

[ok] Process 15838 (hmmscan --noali --notextw --acc -E 1e-09 --cpu 1 /home/anthonyw/anaconda3/db/hmm/HAMAP.hmm /dev/stdi

[ok] Process 15926 (hmmscan --noali --notextw --acc -E 1e-09 --cpu 1 /home/anthonyw/anaconda3/db/hmm/HAMAP.hmm /dev/stdi

[ok] Process 15957 (hmmscan --noali --notextw --acc -E 1e-09 --cpu 1 /home/anthonyw/anaconda3/db/hmm/HAMAP.hmm /dev/stdi

[ok] Process 15986 (hmmscan --noali --notextw --acc -E 1e-09 --cpu 1 /home/anthonyw/anaconda3/db/hmm/HAMAP.hmm /dev/stdi

[ok] Process 16049 (hmmscan --noali --notextw --acc -E 1e-09 --cpu 1 /home/anthonyw/anaconda3/db/hmm/HAMAP.hmm /dev/stdi

[ok] Process 16165 (hmmscan --noali --notextw --acc -E 1e-09 --cpu 1 /home/anthonyw/anaconda3/db/hmm/HAMAP.hmm /dev/stdi

[info] Verification complete

# Phylogenetic inference verification – EnvDst1

[info] Starting replication

[info] Replication complete

[info] Starting verification

[ok] Process 3803 (mkdir reference) executed

[ok] Process 3807 (wget https://www.arb-silva.de/fileadmin/silva_databases/current/Exports/SILVA_138_SSURef_NR99_tax_sil

[ok] Process 3840 (gzip -d SILVA_138_SSURef_NR99_tax_silva.fasta.gz) executed

[ok] Process 3868 (bioawk -c fastx $comment ~ /;Yersinia;/ { print ">" $name " " $comment "\n" $seq } SILVA_138_SSURef_

[ok] Process 3874 (mkdir tree) executed

[ok] Process 3878 (/bin/sh /home/anthonyw/.linuxbrew/bin/mafft --thread 8 ../reference/yersinia.fasta) executed

[ok] Process 3971 (raxmlHPC-PTHREADS -s aligned.fasta -n yersinia -m GTRCAT -T 8 -p 1) executed

[ok] Process 3908 (mktemp -d ./mafft.XXXXXXXXXX) executed

[ok] Process 3909 (cat ../reference/yersinia.fasta) executed

[ok] Process 3910 (tr \r \n) executed

[ok] Process 3916 (/bin/sh /home/anthonyw/.linuxbrew/bin/mafft --thread 8 ../reference/yersinia.fasta) executed

[ok] Process 3919 (/bin/sh /home/anthonyw/.linuxbrew/bin/mafft --thread 8 ../reference/yersinia.fasta) executed

[ok] Process 3923 (/bin/sh /home/anthonyw/.linuxbrew/bin/mafft --thread 8 ../reference/yersinia.fasta) executed

[ok] Process 3926 (/bin/sh /home/anthonyw/.linuxbrew/bin/mafft --thread 8 ../reference/yersinia.fasta) executed

[ok] Process 3929 (/bin/sh /home/anthonyw/.linuxbrew/bin/mafft --thread 8 ../reference/yersinia.fasta) executed

[ok] Process 3932 (/bin/sh /home/anthonyw/.linuxbrew/bin/mafft --thread 8 ../reference/yersinia.fasta) executed

[ok] Process 3935 (/bin/sh /home/anthonyw/.linuxbrew/bin/mafft --thread 8 ../reference/yersinia.fasta) executed

[ok] Process 3938 (/bin/sh /home/anthonyw/.linuxbrew/bin/mafft --thread 8 ../reference/yersinia.fasta) executed

[ok] Process 3957 (/bin/sh /home/anthonyw/.linuxbrew/bin/mafft --thread 8 ../reference/yersinia.fasta) executed

[ok] Process 3969 (cat) executed

[ok] Process 3970 (rm -rf ./mafft.Fwhq03RAA1) executed

[ok] Process 3960 (/home/anthonyw/.linuxbrew/Cellar/mafft/7.305/libexec/mafft/disttbfast -q 0 -E 2 -V -1.53 -s 0.0 -W 6

[ok] Process 3968 (mv hat3.seed hat3) executed

[info] Verification complete

# Phylogenetic inference verification – EnvDst2

[info] Starting replication

[info] Replication complete

[info] Starting verification

[ok] Process 19225 (mkdir reference) executed

[ok] Process 19229 (wget https://www.arb-silva.de/fileadmin/silva_databases/current/Exports/SILVA_138_SSURef_NR99_tax_si

[ok] Process 19261 (gzip -d SILVA_138_SSURef_NR99_tax_silva.fasta.gz) executed

[ok] Process 19290 (bioawk -c fastx $comment ~ /;Yersinia;/ { print ">" $name " " $comment "\n" $seq } SILVA_138_SSURef

[ok] Process 19296 (mkdir tree) executed

[ok] Process 19300 (/bin/sh /home/anthonyw/.linuxbrew/bin/mafft --thread 8 ../reference/yersinia.fasta) executed

[ok] Process 19391 (raxmlHPC-PTHREADS -s aligned.fasta -n yersinia -m GTRCAT -T 8 -p 1) executed

[warning] Process 19391 (raxmlHPC-PTHREADS -s aligned.fasta -n yersinia -m GTRCAT -T 8 -p 1) different version than orig

[ok] Process 19329 (mktemp -d ./mafft.XXXXXXXXXX) executed

[ok] Process 19330 (cat ../reference/yersinia.fasta) executed

[ok] Process 19331 (tr \r \n) executed

[ok] Process 19337 (/bin/sh /home/anthonyw/.linuxbrew/bin/mafft --thread 8 ../reference/yersinia.fasta) executed

[ok] Process 19340 (/bin/sh /home/anthonyw/.linuxbrew/bin/mafft --thread 8 ../reference/yersinia.fasta) executed

[ok] Process 19343 (/bin/sh /home/anthonyw/.linuxbrew/bin/mafft --thread 8 ../reference/yersinia.fasta) executed

[ok] Process 19347 (/bin/sh /home/anthonyw/.linuxbrew/bin/mafft --thread 8 ../reference/yersinia.fasta) executed

[ok] Process 19350 (/bin/sh /home/anthonyw/.linuxbrew/bin/mafft --thread 8 ../reference/yersinia.fasta) executed

[ok] Process 19353 (/bin/sh /home/anthonyw/.linuxbrew/bin/mafft --thread 8 ../reference/yersinia.fasta) executed

[ok] Process 19356 (/bin/sh /home/anthonyw/.linuxbrew/bin/mafft --thread 8 ../reference/yersinia.fasta) executed

[ok] Process 19359 (/bin/sh /home/anthonyw/.linuxbrew/bin/mafft --thread 8 ../reference/yersinia.fasta) executed

[ok] Process 19378 (/bin/sh /home/anthonyw/.linuxbrew/bin/mafft --thread 8 ../reference/yersinia.fasta) executed

[ok] Process 19389 (cat) executed

[ok] Process 19390 (rm -rf ./mafft.YalY9eyyyz) executed

[ok] Process 19381 (/home/anthonyw/.linuxbrew/Cellar/mafft/7.305/libexec/mafft/disttbfast -q 0 -E 2 -V -1.53 -s 0.0 -W 6

[ok] Process 19388 (mv hat3.seed hat3) executed

[info] Verification complete

# VDF configuration

# FASTQ -> FASTA

[entry]

match=\.(fastq|fq)$

ext=.fasta

cmd=fastq_to_fasta -i {input}

# SAM -> Sorted BAM

[entry]

match=\.(sam)$

ext=.bam

cmd=samtools sort {input}

# Clustal -> PHYLIP

[entry]

match=\.(aln)$

ext=.phylip

cmd=./clustal_to_phylip.py {input}

# Headers from FASTA files

[entry]

match=\.(fasta|fa)$

ext=.headers

cmd=grep > {input}

# SAM flagstats

[entry]

match=\.(sam)$

ext=.flagstats

cmd=samtools flagstat {input}

# SAM match abundance

[entry]

match=\.(sam)$

ext=.counts

cmd=/bin/sh -c "grep -v @ {input} | cut -f 3 | sort | uniq -c | sort -n"
